# Avoid equipment graveyards: rigorous process to improve identification and procurement of effective, affordable, and usable newborn devices in low-resource hospital settings

**DOI:** 10.1186/s12887-023-04362-x

**Published:** 2023-11-15

**Authors:** Elizabeth Asma, Megan Heenan, George Banda, Rebecca P. Kirby, Lucky Mangwiro, Claudia Ziegler Acemyan, Kara M. Palamountain, Philip Kortum, Kondwani Kawaza, Z. Maria Oden, Rebecca Richards-Kortum, Technical Collaborative Authorship Group, Alexsandra Brandt, Danica Kumara, Li Jin, Ali Khalid, Cliff Osoo, Nicki Bisceglia, Vince Gate, Maureen Valle, Rowland Mjumira, Abby Chapin, Alyssa Shapiro, Christina Samuel, David Kimmey, M. Grant Belton, Yifan Jack Wang, Jake Johnston, Jessica Anderson, Joseph Bailey, Josh Coyle, Kaede Gordon, Madeleine Tadros Weld, Meaghan Bond, Natalie Mitchell, Sara Liaghati Mobarhan, Sarah Elina Salter, Shababa B. Matin, Sonia E. Sosa Saenz, Sylvie Kalikoff, Taylor Boles

**Affiliations:** 1 Rice University Rice360 Institute for Global Health Technologies, Houston, TX USA; 2 Kamuzu University of Health Sciences, Blantyre, Malawi; 3 Northwestern University Kellogg School of Management, Evanston, IL USA; 4 Department of Psychological Sciences, Rice University, Houston, TX USA; 5 Department of Bioengineering, Rice University, Houston, TX USA; 6 3rd Stone Design, Sausalito, USA; 7 Hatch Technologies, Nairobi, Kenya

**Keywords:** Newborn, Low- and Middle-Income Countries, Medical devices, Technology assessment, Appropriate healthcare technology, User centred design

## Abstract

**Background:**

Millions of newborns die annually from preventable causes, with the highest rates occurring in Africa. Reducing neonatal mortality requires investment to scale hospital care, which includes providing hospitals with appropriate technology to care for small and sick newborns. Expensive medical devices designed for high-resource settings often fail to withstand conditions in low-resource hospitals, including humidity, dust, frequent user turnover, complex maintenance, lack of stable power, or difficulty sourcing expensive consumables. Rigorous evaluation protocols are needed to identify effective, affordable, rugged, and easy-to-use medical devices appropriate for quality hospital-based newborn care in low-resource hospitals.

**Methods:**

We developed an evidence-based technology review process to identify medical devices suitable for small and sick newborn care in low-resource hospitals. The eight-step process consists of: identifying devices needed for effective newborn care; defining Target Product Profiles (TPPs); identifying commercially-available products that may meet TPPs; conducting desk research to evaluate technologies against TPPs; performing technical performance verification testing under laboratory conditions; verifying technical performance after exposure to heat, humidity, dust, and power loss; performing usability evaluations with nurses, and qualifying devices that pass all steps. Devices were purchased, installed, and monitored in newborn wards across Kenya, Malawi, Nigeria, and Tanzania.

**Results:**

Of 271 devices considered, only 45 (16.6%) met corresponding TPPs based on desk research. Thirty-nine were purchased and evaluated in the laboratory; five (12.8%) failed to meet TPPs. Thirty-four products passing laboratory evaluation underwent short-term environmental testing; only one (2.9%) device failed. Thirty-seven products underwent usability testing with 127 clinicians; surprisingly, 14 (37.8%) failed to meet TPPs. Twenty-three products passed all evaluations, and 2457 devices were installed across 65 newborn wards in Kenya, Malawi, Nigeria, and Tanzania. Continuous device monitoring reported minimal device failures, with failed devices typically returned to service within two days, resulting in an average uptime (service days divided by days installed) of 99%.

**Conclusion:**

An evidence-based device selection process can improve procurement of effective, affordable, rugged, usable newborn care devices for low-resource hospitals, and feedback to manufacturers can improve device quality. Similar processes could be adapted beyond newborn care to identify medical devices suitable for implementation in any low-resource setting.

**Supplementary Information:**

The online version contains supplementary material available at 10.1186/s12887-023-04362-x.

## Key findings

**Table:** 

**1. WHAT WAS KNOWN?**
• Reducing neonatal mortality requires medical technology; devices designed for high-resource settings frequently fail in low-resource hospital environments. The aim of this study was to develop rigorous evaluation protocols to identify effective, affordable, rugged, and easy-to-use medical devices appropriate for low-resource settings
**2. WHAT WAS DONE THAT IS NEW?**
• Hundreds of medical devices underwent our evidence-based technology review process, including technical testing, short-term environmental testing, and comparative usability assessments with nurses. Devices were purchased, installed, and monitored in newborn wards across Kenya, Malawi, Nigeria, and Tanzania
**3. WHAT WAS FOUND?**
• Of 271 devices considered, only 45 (17%) met corresponding TPPs based on desk research. Fourteen of 37 devices (38%) failed usability evaluations conducted by 127 clinicians. Twenty-three devices passed all evaluations, and 2457 devices were installed across 65 newborn wards in four sub-Saharan African countries. Ongoing device monitoring reported failed devices typically returned to service within two days
**4. WHAT DOES THIS MEAN?**
• Research and development of newborn devices continues to be urgently needed to meet TPPs. Ensuring medical devices are easy to use is a key area where device developers can make improvements. Engaging global agencies is critical to guarantee appropriate technologies are on national procurement lists. Similar processes could be adapted beyond newborn care to identify medical devices suitable for implementation in any low-resource setting

## Background

Every year worldwide, over 2.3 million newborns die, and 30 million small and sick newborns require hospital care [1]. Indeed, neonatal conditions are a leading cause of death in low-income countries [2]. The highest rates of newborn death are in Africa [1], where the rate of progress to improve newborn survival is slowest. More than one million African newborns die annually, the majority from preventable causes [1]. In the face of this challenge, the world has pledged, for the first time, to end preventable newborn deaths. Sustainable Development Goal (SDG) 3.2 aims for all countries to reduce neonatal mortality rates below 12 per 1000 livebirths by 2030 [3]. However, only one country in sub-Saharan Africa is on track to achieve the SDG for newborn survival. At current rates of progress, sub-Saharan Africa will be the last global region to achieve this goal. Some African countries will meet SDG 3.2 more than 100 years too late [4].

Historically, reducing neonatal mortality below the SDG target requires investment to scale hospital care during labour, delivery, and the first week of life, especially for small and sick babies [5–8]. In the 1970s, the US and UK scaled national programs of hospital-based small and sick newborn care (SSNC) [9, 10]; neonatal mortality rates dropped below 15/1000 for the first time [11]. To help achieve SDG 3.2 for newborns, the *Every Newborn* *Action Plan* (ENAP) lays out coverage targets for achieving high-quality antenatal care, essential childbirth care, postnatal care, and in-patient care for small and sick newborns, with equity in all countries [12]. ENAP coverage target 4 specifically calls for 80% of districts to have at least one unit equipped to provide WHO level-2 in-patient SSNC, including provision of respiratory support with continuous positive airway pressure (CPAP) [13].

**Fig. 1:**
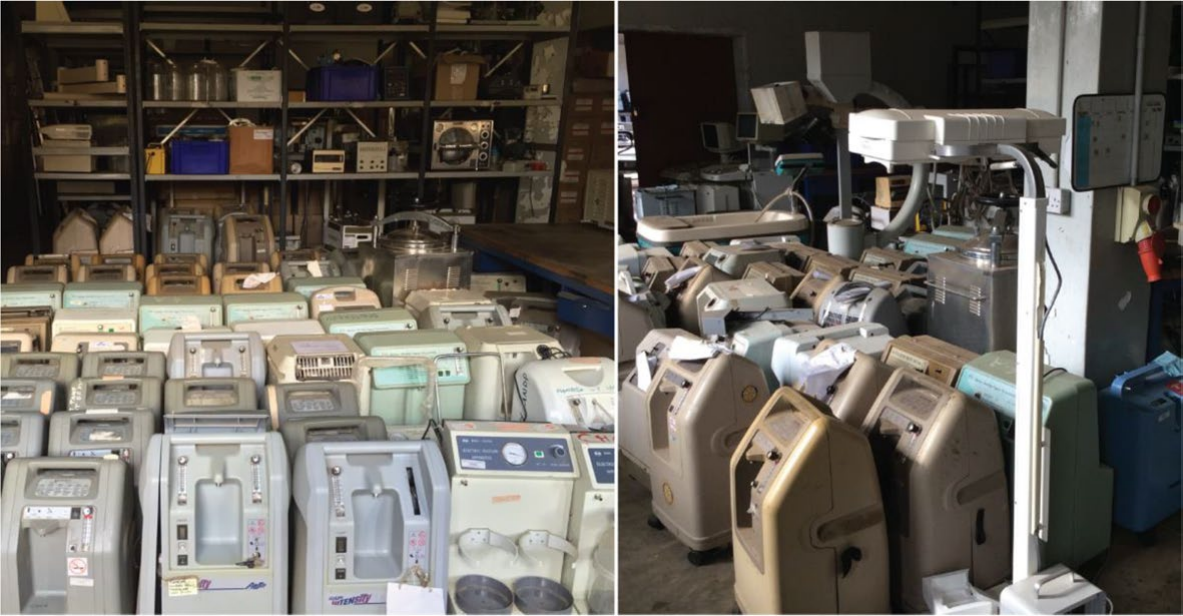
Medical equipment graveyards in low-resource hospitals. Medical devices designed for use in high-resource settings often fail when used in harsh environmental conditions found in low-resource hospitals; because user instructions or spare parts are not accessible, broken devices remain in equipment graveyards like those pictured here in Malawi. Therefore, rigorous evaluation protocols are needed to identify medical devices that are effective, affordable, rugged, and easy to use in low-resource settings. (Photo credit: Brandon Martin, 2016)

Unfortunately, most African hospitals do not have the resources to provide level-2 care plus CPAP for small and sick newborns [6]. Many units lack functional equipment needed to provide level-2 care, including medical devices that monitor, prevent, and treat respiratory distress, infection, hypothermia, neonatal jaundice, and other neonatal conditions. Additionally, many commercially available medical devices to support level-2 neonatal care were designed for use in high-resource settings; they are too costly for low-resource settings and are not designed to withstand the harsh environmental conditions in low-resource hospitals, such as dust, humidity, heat, and electrical power fluctuations [14, 15]. Equipping hospitals in low-resource settings with devices designed for high-resource settings often results in equipment graveyards (Fig. 1), stockpiles of expensive technologies that quickly fail due to harsh environmental conditions, frequent user turnover, complex maintenance requirements, or lack of stable infrastructure, such as line voltage fluctuations [16–18]. Moreover, it can be difficult to source expensive consumables or spare parts needed to sustain the use of such devices.

To meet and sustain ENAP coverage target 4 [13], there is an important need to equip hospitals in low-resource settings with newborn technologies that are effective, affordable, rugged, and easy to use by nurses and maintain by engineers. Target Product Profiles (TPPs) have recently been established to define the setting, target user, and range of performance characteristics for 15 types of newborn care devices for use in low- and middle-income country settings [19]. However, procurement officers in low-resource settings need publicly available information about which commercially available devices meet these TPPs. Similarly, product developers need information about remaining market gaps as well as areas where new devices are needed to meet the TPPs [20–24].

This paper aims to describe the development and implementation of a process to evaluate and qualify whether commercially available medical technologies for SSNC settings meet TPPs for low-resource hospitals. This information can help procurement officers ensure they obtain newborn care equipment that is effective, affordable, usable, and will last. It can help device manufacturers understand points of failure and how devices designed for use in high-resource settings can be adapted and improved to serve broader global needs. Without efforts to identify, source, and sustain qualified technologies for SSNC in hospitals, one million African newborns will continue to die from preventable causes each year.

## Methods

**Fig. 2:**
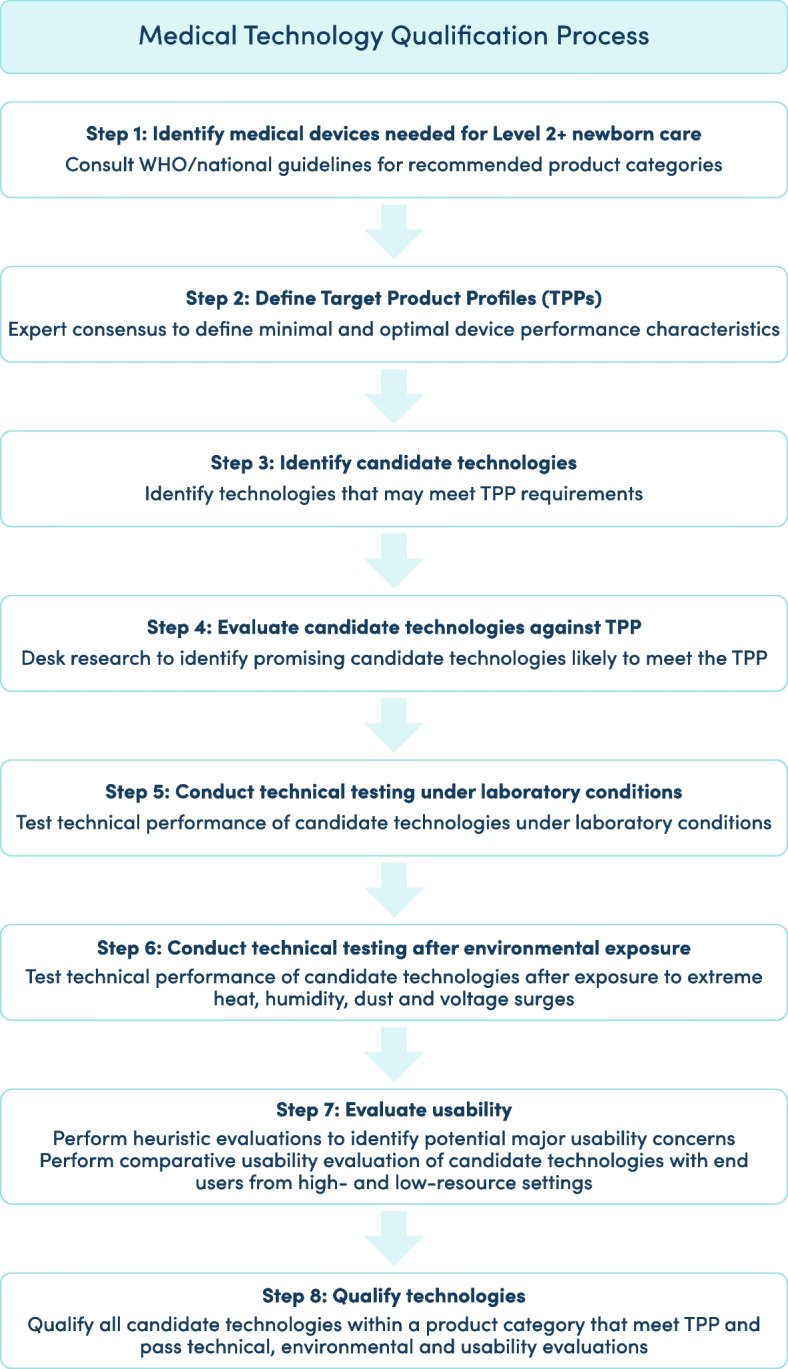
Process to qualify technologies for newborn care in low-resource hospitals. Level 2+  = level-2 newborn care plus continuous positive airway pressure

Described here is an evidence-based technology review process designed to identify and qualify medical devices suitable for use in low-resource hospitals. A step-by-step overview of the device qualification process is shown in Fig. 2.

Briefly, the eight-step process consists of: (1) Identifying types of medical devices needed for care in a particular setting; (2) Defining TPPs for each device product category; (3) Identifying commercially available products that may meet the TPPs; (4) Conducting desk research to evaluate candidate technologies against the TPPs; (5) Performing laboratory testing to verify technical performance under laboratory conditions; (6) Performing testing to verify technical performance after exposure to harsh environmental conditions; (7) Performing usability evaluations with target users, and (8) Qualifying devices that pass all evaluation process steps. A standardised report card summarises the evaluation results for candidate technologies within each product category.

Below, we describe the process used to evaluate and qualify medical technologies for level-2 SSNC, including respiratory support with CPAP in low-resource hospitals.

### Step 1: Identify medical devices needed for level-2 newborn care plus CPAP

WHO guidelines for improving the quality of care for small and sick newborns in health facilities [25, 26] and national guidelines for care of small and sick newborns in Kenya, Malawi, Nigeria [27], and Tanzania were reviewed to identify the types of medical devices commonly recommended to provide hospital-based level-2 care for small and sick newborns including respiratory support with CPAP. The types of devices identified include 14 product categories for level-2 newborn care plus respiratory support: syringe pump, bilirubinometer, phototherapy, glucometer, haemoglobinometer, CPAP, flow splitter, oxygen concentrator, continuous pulse oximeter, suction pump, radiant warmer, continuous temperature monitor, conductive warmer, and respiratory rate/apnoea monitor.

### Step 2: Define Target Product Profiles (TPPs)

Global stakeholders were consulted to develop a TPP that defines optimal and minimal performance characteristics for each type of medical device for small and sick newborn care [28]. The TPP development process is documented in a separate publication [19]. The characteristics include target operator, target population, target setting, regulatory approval, relevant technical characteristics (e.g., for diagnostic devices: accuracy, range, precision, response time, type, and required volume of sample), consumable storage requirements, frequency of calibration, therapeutic dose delivered for therapeutic devices (including range and accuracy), size, mobility, inclusion of warning alarms, requirements for proprietary or non-proprietary accessories or consumables, cost (including ex-works equipment and consumable costs), power requirements (including line voltage requirements, inclusion of battery backup, battery life), maintenance requirements, and availability of user instructions/training.

### Step 3: Identify candidate technologies

A comprehensive search was conducted to identify commercially available medical devices within each product category. Candidate technologies were identified by consulting international device resources, including a publicly available landscape of newborn care devices updated every six months [29], recommendations from newborn care experts, consultations with leading manufacturers and distributors of newborn care devices, and WHO Compendia of Innovative Technologies for Low-Resource Settings [30].

### Step 4: Evaluate candidate technologies against TPP

Desk research was performed for each candidate technology to compare its operational and performance characteristics as described in manufacturer package inserts and publicly available materials to the minimal and optimal values outlined in the TPPs.

**Fig. 3:**
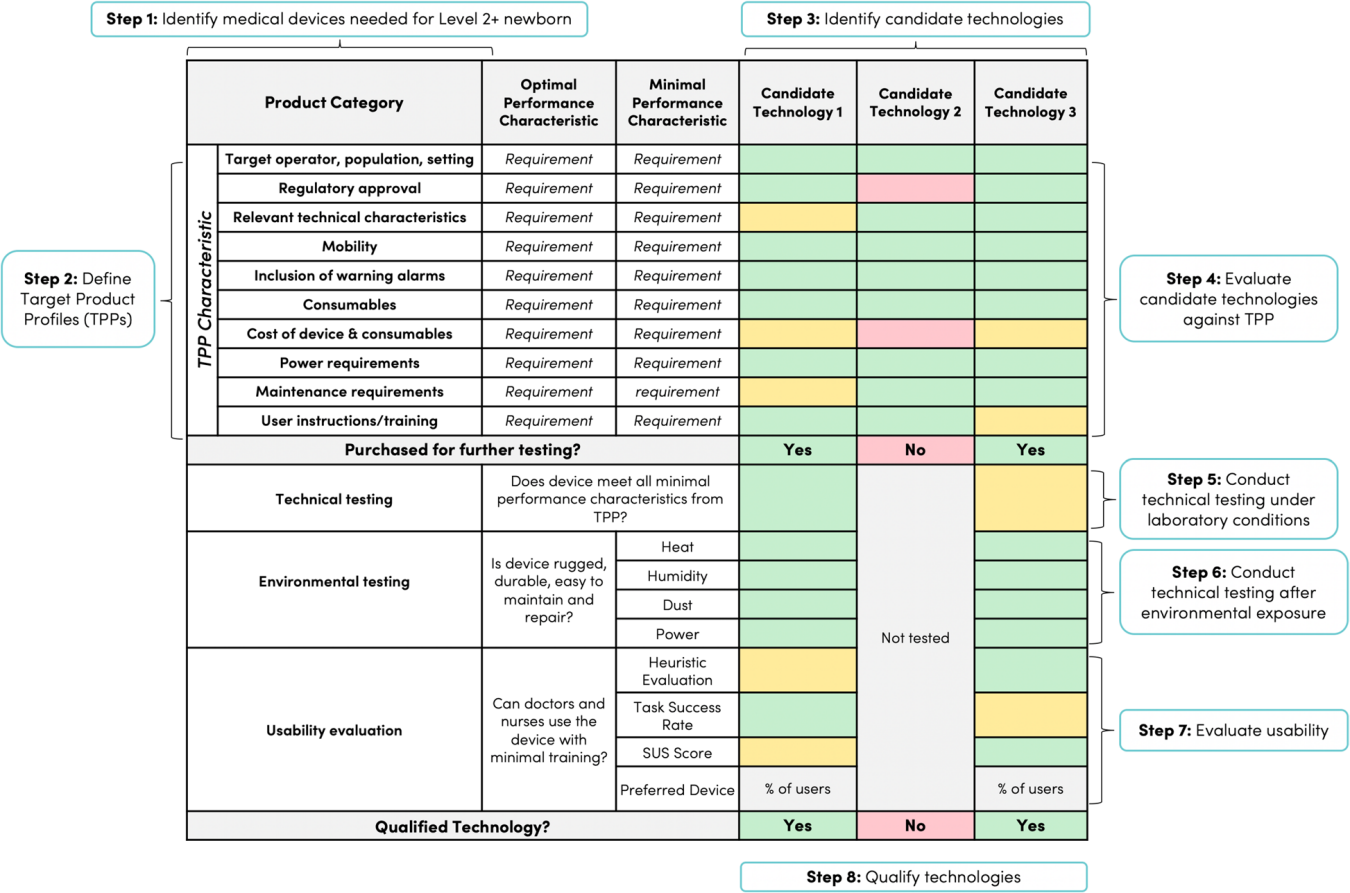
Generic product category report card. For each product category, a standardised product report card is used to document evaluation results at each of the eight steps of the medical technology qualification process. Cells are coloured green if the candidate technology meets optimal TTP, environmental, or usability requirements, yellow if the technology meets minimal TPP, environmental, or usability requirements, and red if it does not meet either specification. Technologies that meet all TPP requirements and pass environmental and usability evaluations are designated as qualified for use in low-resource hospitals. Level 2+ = level-2 newborn care plus continuous positive airway pressure. * Abbreviations:* *SUS* System Usability Survey, *TPP* Target Product Profile

A product report card was developed to summarise and compare evaluation results of candidate technologies within each product category. Each row of the report card corresponds to a TPP characteristic, and each column of the report card corresponds to a candidate technology. As shown in Fig. 3, cells in the report card were coloured green if the candidate technology met the optimal TPP specification, yellow if the technology met the minimal TPP specification, and red if it did not meet either. The most promising candidate technologies were identified based on the product report card. Two units of each promising candidate technology were purchased for further testing and evaluation.

### Step 5: Technical testing

Technical testing protocols were developed for each product category to measure whether a candidate technology met the minimal or optimal TPP characteristics. Table 1 summarises the major components of each test protocol for every product category, and full testing protocols are available (see Additional file 1).

**Table 1:** Summary of characteristics evaluated in technical testing by product category

Product category	Quantitative characteristics evaluated	Qualitative characteristics evaluated	Required evaluation tools
Syringe Pump	• Benchtop measurement accuracy across range of flow rates • Battery life • Device weight	• Includes occlusion detection alarm • Accepted syringe brands • Accepted syringe sizes • Includes visual and auditory alarms	• Syringes (various brands, sizes) • Infusion line • Scale • Water
Bilirubinometer	• Accuracy • Precision • Linear range • Volume of sample required	• Units displayed • Requires calibration materials • Transport and storage limitations; temperature, humidity, altitude • Requires consumables	• *Reichert UNISTAT* gold standard • Centrifuge • Stock solution of bilirubin • Blood (spiked to test range of bilirubin concentrations)
Phototherapy	• Effective treatment area • Irradiance at standard, intensive levels • Peak wavelength • Power consumption	• Bulb type, lifetime • Ease of replacing bulbs • Includes light meter	• Spectrometer • Watt meter
Glucometer	• Accuracy • Precision • Linear range • Volume of sample required	• Units displayed • Requires calibration materials • Transport and storage limitations; temperature, humidity, altitude • Requires consumables	• *YSI 2300* *STAT PLUS* benchtop analyser • Centrifuge • Standard 917c D-Glucose • Blood (spiked to test range of glucose concentrations)
Haemoglobinometer	• Accuracy • Precision • Linear range • Volume of sample required	• Units displayed • Requires calibration materials • Transport and storage limitations; temperature, humidity, altitude • Requires consumables	• *Beckman Coulter AcT Diff II* benchtop analyser • Centrifuge • Blood (diluted or concentrated to test range of haemoglobin concentrations)
CPAP	• Oxygen flow capability • Pressure • Total (blended) flow	• Provides humidification • Include alarms for low flow, low pressure, power loss • Requires proprietary consumables	• Gas analyser • Oxygen source
Flow Splitter	• Air flow per outlet • Independent ability to control flow per outlet	• Number of outlets • Visual indicators for flow rate	• Flow meter • Oxygen source
Oxygen Concentrator	• Flow rate • Time to reach 95% performance • Oxygen purity • Sound level • Power efficiency • Power consumption	• Includes alarms for high temp, low flow, high/low pressure • Visual indicators show device status • Mobility • Filter cleaning • Decontamination	• Oxygen analyser • Oxygen cylinder (100% O2) • Decibel sound meter • Watt meter • Scale
Continuous Pulse Oximeter	• Pulse rate range, accuracy • SpO_2_ range, accuracy • Battery life	• Alarm limits adjustable • Includes visual and auditory alarms • Requires consumables • Ease of decontamination	• Patient simulator • SpO2 finger simulator
Suction Pump	• Pressure range • Sound level	• Bottle capacity • Includes fail-safe to protect pump • Ease of decontamination • Requires consumables	• Negative pressure gauge • Decibel sound meter
Radiant Warmer	• Temperature probe accuracy • Temperature stability • Time to indicate accurate temperature • Uniformity • Power consumption	• Alarm limits for hypothermia and hyperthermia adjustable • Includes visual and auditory alarms • Requires consumables • Ease of decontamination • Mobility	• Water bath • Reference thermometer • Aluminium discs • Timer • Watt meter
Continuous Temperature Monitor	• Temperature accuracy • Time to indicate accurate temperature	• Alarm limits for hypothermia and hyperthermia adjustable • Includes visual and auditory alarms • Requires consumables • Ease of decontamination	• Water bath • Reference thermometer • Timer
Conductive Warmer	• Temperature accuracy • Time to indicate accurate temperature • Uniformity • Power consumption	• Temperature control, either baby or manual • Include visual and auditory alarms • Requires consumables • Ease of decontamination	• Water bath • Reference thermometer • Watt meter
Respiratory Rate/Apnoea Monitor	• Respiratory rate accuracy, range • Apnoea detection	• Includes visual and auditory alarms • Easy to clean	• NeoNatalie^TM^ newborn simulator • Timer

*Abbreviations*: *CPAP* Continuous positive airway pressure, *O*_*2*_ oxygen, *SpO*_*2*_ oxygen saturation

Three testers independently evaluated the technical performance of two units of each candidate technology. Technical results from each evaluation were recorded by device serial number. Testing was performed under ambient environmental conditions in a research laboratory. Results were compared to TPP specifications; to pass, each device had to meet or exceed the minimal TPP performance characteristic when evaluated by each tester. Results of laboratory testing were documented in product report cards.

### Step 6: Environmental testing

The suitability of candidate devices for the operating environments of low-resource hospital settings was established by evaluating technical performance following exposure to harsh environmental conditions during testing. Environmental exposure protocols were developed to mimic sustained exposure of devices to conditions of high heat, humidity, dust, and line voltage fluctuations; Table 2 summarises exposure protocols. Detailed environmental testing protocols and technical performance metrics by product category are available (see Additional file 2).

**Table 2:** Summary of environmental exposure conditions and relevant standards

Test condition	Description	Relevant standard
High temperature	Constant device operation under high heat; 50 °C and relative humidity < 50% for 16 h	IEC 60068–2-2
High humidity	Constant device operation under high humidity; relative humidity 95% cycling between 25 °C and 40 °C for 48 h, held at each temperature twice for 12 h	IEC 60068–2-30
Dust exposure	Recirculated 400 g/m^2^ dust particles < 75 μm for 4 h	IEC 60069–2-68
Line voltage fluctuations and power failure	Varied line voltage by ± 8%, ± 12%, and simulated total power failure	IEC 61000–4-14

*Abbreviations*: *h* hours, *IEC* International Electrotechnical Commission

Technologies were powered on throughout the duration of exposure to harsh environmental conditions, but power was cycled between environmental exposures. One tester repeated technical testing of one unit of each candidate technology after exposure to harsh environmental conditions; to pass, the device had to meet or exceed the minimal TPP performance characteristic following exposure.

Candidate technologies were first exposed to heat in an environmental test chamber in accordance with International Electrotechnical Commission (IEC) 60068–2-2. The temperature was held at 50 °C and relative humidity < 50% for 16 hours (h). This exceeds the maximum value observed at the intended hospital sites, where temperature monitoring indicated that neonatal ward temperatures frequently reached temperatures exceeding 40 °C. After being returned to ambient temperature, technical performance metrics were measured and documented in product category report cards.

Candidate technologies were then exposed to high humidity in an environmental test chamber in accordance with IEC 60068–2-30. Relative humidity was held at 95% while the temperature was cycled between 25 °C and 40 °C for 48 h; each temperature cycle was 12 h in duration. These conditions mimic those at the intended hospitals where constant humidity and temperature monitors documented relative humidity values ranging from 35 to 100%, while ambient temperature values in the ward cycled between values corresponding to daily outdoor high and overnight low temperatures. After being returned to ambient conditions, technical performance metrics were measured and documented in product category report cards.

Candidate technologies were then exposed to dusty conditions in a dust chamber in accordance with IEC 60069–2-68, which recirculated 400 g/m^2^ fine dust particles < 75 μm (Arizona test dust A3 medium) for 4 h. Dust particle size analysis was performed on dust collected from a ward in Malawi to determine a representative dust particle profile. The total volume of devices took up < 25% of the test chamber volume, and the total base area remained < 50% horizontal working surface. Devices were placed within the chamber in a manner that ensured they did not shield each other from dust. Relative humidity inside the chamber was kept at 35–40%, and dust was allowed to settle for two hours prior to opening the chamber. Dust was lightly wiped off devices to make them accessible to operate, and then technical performance metrics were measured and documented in product category report cards. This dust exposure protocol was designed to simulate over 1.5 years of dust exposure measured without preventative maintenance or cleaning based on volume of dust collected over time in a central hospital in Malawi. Bilirubinometers, glucometers, haemoglobinometers, and pulse oximeters were excluded from dust testing since they are small, portable devices that are frequently cleaned between use.

Candidate technologies that require mains power or have an alternating current (AC) wall adapter for charging were exposed to line voltage fluctuations and conditions simulating total power failure. These three power failure conditions represent power scenarios commonly observed at various hospital sites, mild fluctuations to line voltage (8%), extreme fluctuations to line voltage (12%) and complete power failure. A programmable AC power source (BK Precision Model 9805) was used to create line voltage sags and surges of ± 8% and ± 12% voltage in accordance with IEC 61000–4-14 Class 2 and Class 3 with nominal voltage 220 V. To simulate total power failure, line voltage was cycled between 220 and 0 V in accordance with IEC 61000–4-14 Class X. After being returned to nominal line voltage conditions of 220 V, technical performance metrics were measured and documented in product category report cards.

### Step 7: Usability evaluation

Domain-specific heuristics allow evaluators to capture usability issues specific to the intended use environment [31]. Three evaluators assessed candidate technologies and identified potential usability concerns using domain-specific heuristics, which accounted for usability needs specific to low-resource settings, including cleanability, maintainability, ease of repair, low workload, minimising discomfort, and access to baby [32].

Heuristic results were used to eliminate candidate technologies with potentially catastrophic usability concerns. Candidate technologies with more than one identified heuristic violation of severity rating 4 per Nielsen’s heuristic severity ratings [33] were eliminated and marked red in the heuristic evaluation section of the report card. All candidate technologies with one or less heuristic violation of severity rating 4 advanced to usability testing with clinicians and were marked as green in the heuristic evaluation section of report cards. Heuristic severity ratings of candidate technologies are documented in product report cards, and detailed methods are available (see Additional file 3) adapted from heuristic applications on medical devices [34]. Streamlined cognitive walkthroughs were conducted together by evaluators after documenting heuristics to identify or clarify any deviations from a typical device procedure [35].

Because syringe pumps are known to have major usability concerns [36], the heuristics and cognitive walkthrough portion of Step 7 was performed prior to Steps 5 and 6 for devices in the syringe pump category to save time and resources that might be devoted to evaluating technical performance of candidate technologies likely to be eliminated by usability concerns.

For all devices within a product category, comparative usability testing was then performed with two groups of end-users. Detailed usability evaluation protocols (see Additional file 4) were developed following best practices in the field of usability [37]. Usability testing was first performed with representative users in Houston, Texas, including medical students, nurses, and physicians. Usability testing was then performed with users in Blantyre, Malawi, including nurses and physicians working at both central and district hospitals in Malawi. All participants were recruited as part of protocols approved by the Rice University Institutional Review Board and the University of Malawi College of Medicine Research Ethics Committee. All surveys and interviews were completed in English, a language spoken by participants across both countries. After watching a brief instructional video, users were asked to complete a small series of typical and/or critical tasks for each candidate technology within a product category (Table 3).

**Table 3:** Summary of usability tasks performed by product category

Product category	Tasks
Syringe Pump	• Begin an infusion of 10 mL of fluid at a rate of 2 mL/hr
	• Explain what the occlusion alarm means and resume the infusion
Bilirubinometer	• Calibrate the reader
	• Measure and report the bilirubin level in the provided blood sample
Phototherapy	• Set up and use the device in high intensity mode
Glucometer	• Measure and report the glucose level in the provided blood sample
Haemoglobinometer	• Measure and report the haemoglobin level in the provided blood sample
CPAP	• Set up the CPAP with all the provided tubing so it has a pressure of 6 cm of water
	• Turn on the CPAP and adjust the total flow rate to between 4 and 6 LPM, with an oxygen percentage from 30 to 50%
	• Assemble the nasal prong interface and connect it to the baby and the CPAP
Flow Splitter	• Set up and use the device to deliver air to three babies, each requiring a different flow rate
Oxygen Concentrator	• Set up and use the device to treat one infant with a flow of 2.0 LPM
	• Remove and wash any required filters
Pulse Oximeter (Continuous)	• Measure and report the simulated heart rate and SpO2 level
	• Set alarm limits of 50–150 for heart rate and 88–100 for Sp02
Suction Pump	• Set up the device, so it is ready to be used with a patient
	• Turn on the device and transfer fluid into the suction container
Radiant Warmer	• Prewarm the device for the required amount of time
	• Place the baby in the bed. Set the device so that it will begin to adjust the baby’s temperature to 37 °C automatically
	• Read and report the baby’s current temperature
	• Explain what the power failure alarm means and fix the cause of the alarm
Temperature Monitor (Continuous)	• Set up the device
	• Secure monitor on patient
	• Identify the source and report the cause of the high temperature alarm
Conductive Warmer	• Set up the device for thermal treatment at 36.5 °C
	• Provide thermal treatment to baby
	• Report the cause of the power failure alarm
Respiratory Rate/Apnoea Monitor	• Set up and use the device to monitor on infant
	• Explain what the apnoea alarm means and fix the cause of the alarm

*Abbreviations*: *mL* millilitres, *hr* hour, *cm *centimetres, *LPM* litres per minute, *CPAP* continuous positive airway pressure, *SpO2* oxygen saturation

A total of 127 users completed usability evaluations across 11 product categories, including 51 participants from Houston and 76 participants in Malawi. Demographics of participants are summarised in Table 4.

**Table 4:** Demographics of participants in usability evaluations

	**Houston (*n* = 51)**	**Malawi (*n* = 76)**
Gender	19 females, 32 males	56 females, 20 males
Average age [range]	29 years [23–40]	35 years [27–65]
Average length of experience in medical profession [range]	4 years [1–10]	12 years [3–33]
Average ranking: “I use technology daily.” 1 – strongly disagree; 5 – strongly agree	4.95 ± 0.2	4.32 ± 0.5

Products in each category were evaluated by an average of six users, with some users evaluating more than one product category as time allowed. In Houston, 18 users evaluated products in more than one product category. In Malawi, 14 users evaluated products in more than one product category. The order of candidate technologies was randomised among participants to account for ordering bias.

Usability assessment results were documented using the International Organization for Standardization (ISO) 9241–11 suggested metrics: efficiency (the time required to complete a task), effectiveness (the percentage of users able to complete each task successfully), and user satisfaction [38]. A modified system usability survey (SUS) [39] was used to capture user satisfaction. The SUS is a validated survey to capture subjective user satisfaction with a device [40]. Users were also asked to identify their preferred device within each product category. Exit interviews were conducted with users to understand challenges or concerns about clinical use of the evaluated technologies, including the potential for alarm fatigue, concerns about consumables, or the ability to clean a device properly. Results of usability testing were documented in product report cards, with SUS scores identified as ≥ 70 optimal (green), < 70 and ≥ 50 minimal (yellow), and < 50 below minimal (red) to align with adjective ratings that describe SUS scores [41].

### Step 8: Qualify technologies

Product report cards were used to assess and document the suitability of each candidate technology for use within a low-resource hospital. Candidate technologies that met all minimal TPP requirements and additionally passed technical, environmental and usability evaluations were identified as qualified to provide effective newborn care in low-resource settings.

Feedback and test results were provided to manufacturers of devices that were evaluated but did not pass the qualification process; in some cases, this led to collaborative discussions that resulted in efforts to improve product performance.

#### Field evaluation of qualified technologies

Qualified technologies from eight product categories (phototherapy lights, glucometer, CPAP, flow splitter, oxygen concentrator, pulse oximeter, suction pump, radiant warmer) were purchased and installed in 65 newborn wards across Kenya, Malawi, Nigeria, and Tanzania, starting in October 2019. Equipment was installed by local equipment dealers in Tanzania, Kenya, and Nigeria and by Newborn Essential Solutions and Technologies (NEST360) staff in Malawi. Device up-time, defined as the number of days the device is functional compared to the number of days the device is installed and available for use, device failures, and time to respond to failures, were monitored by local equipment dealers or program staff. Feedback regarding the performance history of qualified technologies implemented in hospitals in low-resource settings was provided to manufacturers where relatively minor changes could significantly improve device performance.

## Results

Fourteen types of medical devices commonly used in level-2 newborn care, plus respiratory support with the provision of CPAP, were identified based on WHO and national guidelines. Product categories included syringe pump, bilirubinometer, phototherapy, glucometer, haemoglobinometer, CPAP, flow splitter, oxygen concentrator, continuous pulse oximeter, suction pump, radiant warmer, continuous temperature monitor, conductive warmer, and respiratory rate/apnoea monitor. TPPs for each of these product categories are publicly available, hosted by UNICEF [28].

We evaluated a total of 271 medical devices across 14 product categories. All 14 product category report cards are available (see Additional file 5). Table 5 summarises the number of candidate technologies identified and evaluated at each step of the process.

**Table 5:** Number of technologies at each step of the qualification process

Steps 1 and 2	Step 3	Step 4		Step 5		Step 6								Step 7		Step 8
**Product category**	**Identified candidate technologies**	**Desk research met TPP**		**Technical testing**		**Environmental testing**								**Usability testing**		**Qualified devices**
						Heat		Humidity		Dust^a^		Power^b^				
		# For review	# Met TPP	# Test	# Pass	# Test	# Pass	# Test	# Pass	# Test	# Pass	# Test	# Pass	# Test	# Pass	Total
Syringe Pump	21	21	6	3	2	2	2	2	2	2	2	2	2	6	1	1
Bilirubinometer	12	12	3	3	3	3	3	3	3	N/A		3	3	3	2	2
Phototherapy	17	17	4	3^d^	3	3	3	3	3	3	3	3	3	3	3	3
Glucometer	13	13	3	3	3	3	3	3	3	N/A		2^f^	2	3	2	2
Haemoglobinometer	20	20	7	7	5	5	5	5	5	N/A		3^f^	3	5	2	2
CPAP	18	18	3	3	3	3	3	3	3	3	3	3	3	3	2	2
Flow Splitter	4	4	3	3	3	3	3	3	3	3	3	N/A		3	3	3
Oxygen Concentrator	24	24	4	3^e^	3	3	3	3	3	3	3	3	3	3	3	3
Pulse Oximeter (Continuous)	25	25	6	6	5	5	5	5	5	N/A		5	5	5	2	2
Suction Pump	7	7	3	3	2	2	2	2	2	2	2	2	1	1	1	1
Radiant Warmer	13	13	3	2^e^	2	2	2	2	2	2	2	2	2	2	2	2
Temperature Monitor (Continuous)^c^	30	30	0	—	—	—	—	—	—	—	—	—	—	—	—	0
Conductive Warmer^c^	31	31	0	—	—	—	—	—	—	—	—	—	—	—	—	0
Respiratory Rate/Apnoea Monitor^c^	36	36	0	—	—	—	—	—	—	—	—	—	—	—	—	0
**Total**	271	271	45	39	34	34	34	34	34	18	18	28	27	37	23	23

^a^Devices excluded from dust testing; small, portable devices are frequently cleaned between patients

^b^Devices excluded since does not require power

^c^No devices currently commercially available meet TPP

^d^One device did not advance due to product discontinued by manufacturer

^e^One device did not advance due to two comparable products identified from same manufacturer, advanced lower-cost model from manufacturer

^f^Devices without rechargeable option through mains power were excluded from power testing

*Abbreviations*: *TPP* Target Product Profile, *CPAP* Continuous positive airway pressure, *N/A* not applicable

**Fig. 4:**
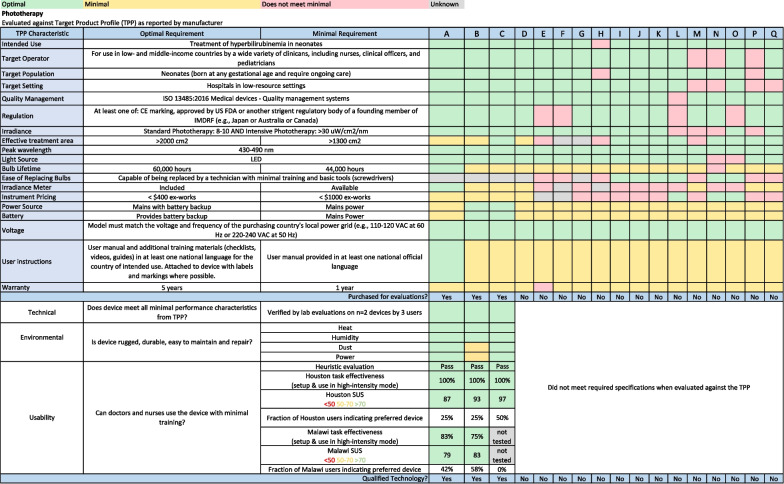
Report card for the product category of phototherapy lights. Most commercially available phototherapy lights were unsuitable for use in low-resource settings due to price, inability to replace lightbulbs, and lack of stringent regulatory body approval. Candidate technology D was not purchased for evaluation due to the product being discontinued. Three phototherapy lights from two manufacturers that passed technical, environmental, and usability testing were designated as qualified for use in low-resource settings. * Abbreviations:* *TPP* Target Product Profile, *ISO* International Organization for Standardization, *IMCAF* International Medical Device Regulators Forum, *LED* light-emitting diode, *VAC* Volts Alternating Current, *Hz* hertz, *SUS* System Usability Survey

Of the 271 devices considered, only 45 (16.6%) met the corresponding TPPs based on desk research. Common reasons devices did not meet TPPs include high cost, the required use of proprietary consumables, lack of regulatory approval, or lack of regulatory approval for use with newborns. We did not identify any qualified devices for three product categories (continuous temperature monitor, conductive warmer, and respiratory rate/apnoea monitor) because no commercially available candidate technologies met the TPP in those three product categories. We purchased 39 of these products and evaluated their performance in the laboratory; five (12.8%) failed to meet the TPPs. Thirty-four products passing laboratory evaluation were subject to short-term environmental testing; only one (2.9%) device failed. Thirty-seven products underwent usability testing; of these, 14 (37.8%) failed to meet the TPP. Notably, five of the six syringe pumps evaluated did not meet the TPP for usability. A total of 23 technologies in 11 of 14 product categories passed all evaluation stages and were qualified for newborn care in low-resource settings. Figure 4 shows the report card for the product category of phototherapy lights.

**Fig. 5:**
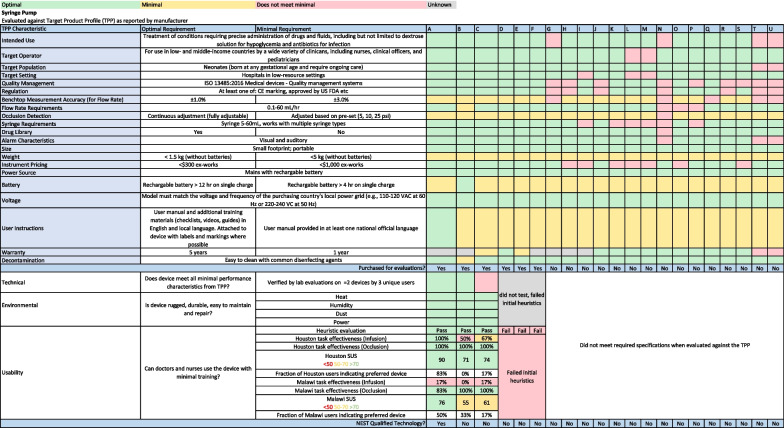
Report card for the category of syringe pump. Most syringe pumps were found to be unsuitable for use in low-resource settings due to a requirement for proprietary syringes and extreme difficulty with usability. One syringe pump was identified that passed technical, environmental, and usability evaluations and designated as qualified for use in low-resource settings. *Abbreviations: TPP* Target Product Profile, *ISO* International Organization for Standardization, *psi* pounds per square inch, *mL* millilitres, *kg* kilogram, *hr* hour, *VAC* Volts Alternating Current, *Hz* hertz, *SUS* System Usability Survey

We identified 17 candidate phototherapy devices. Of these, desk research indicated that four appeared to meet the TPP characteristics. The manufacturer discontinued one of the four, and the remaining three were purchased for testing. Common reasons the other 14 phototherapy devices did not meet the TPP include high cost, difficulty replacing lightbulbs, and lack of regulatory approvals. All three candidate phototherapy devices passed technical, environmental, and usability testing and were designated as qualified for newborn care in low-resource settings. Figure 5 shows the report card for the product category of syringe pump.

We identified 21 candidate syringe pumps. Of these, desk research indicated six appeared to meet the TPP characteristics; these were purchased for testing. The other 15 syringe pumps did not meet the TPP, most commonly due to the requirement of proprietary syringes and instrument pricing. Of the six purchased syringe pumps, only three passed the heuristic usability evaluation, and these were subject to technical, environmental, and usability testing. Two passed technical testing, but only one passed usability testing and was identified as qualified for newborn care in low-resource settings. Major usability concerns identified during usability testing included that a significant fraction of users could not correctly start an infusion with the desired volume and/or flow rate.

### Field evaluation of qualified technologies

From October 2019 until December 2022, 2457 devices from eleven product categories were procured by a global equipment dealer and installed in 65 newborn wards at tertiary and secondary hospitals in Kenya, Malawi, Nigeria, and Tanzania covering a variety of staffing levels, power quality and environmental conditions. This included 53 syringe pumps, 32 bilirubinometer, 288 phototherapy lights, 22 glucometers, three haemoglobinometers, 416 CPAP machines, 234 flow splitters, 502 oxygen concentrators, 443 pulse oximeters, 237 suction pumps, and 227 radiant warmers. Devices in Kenya, Nigeria, and Tanzania were installed by a local equipment dealer and maintained by project and local biomedical staff. Devices in Malawi were installed by project staff and maintained by project and local biomedical staff. A health technology management system was used by local dealers and local biomedical engineering technicians to report the functionality of devices. The number of device failures reported to the global equipment dealer was 337, and local equipment dealers, on average, were able to return failed devices into service within two days. From installation to December 2022, the average up time of equipment, defined as days in service divided by days since installation, is 99%.

Table 6 summarises field evaluation data by product category.

**Table 6:** Summary of field evaluations by product category

Product category	Reported device failures	Response and resolution
Syringe Pump	No accumulated data, devices introduced in 2022	
Bilirubinometer	No accumulated data, devices introduced in 2022	
Phototherapy	10 devices reported low irradiance	Manufacturer provided repair guide to clean, eliminate loose terminal connections
Haemoglobinometer	No accumulated data, devices introduced in 2022	
CPAP	Flowmeter oxygen tubing dislodging under pressure if connected when flowmeter partially closed Bottle straw deteriorating, forming cracks	Flowmeter oxygen tube secured with a zip tie, manufacturer provided spare zip ties with new devices Manufacturer redesigned bottle straws and sent replacement parts
Flow Splitter	No flow outlet, crashed bobbin, damaged flowmeter control knob	Replacement parts stocked to replace broken or damaged parts. User training to reinforce gentle handling of the device, as most breakages are user errors
Oxygen Concentrator	Zeolite leakage from sieve beds	Manufacturer provided filter papers for sieve beds to prevent Zeolite leakages
Pulse Oximeter (Continuous)	SpO2 board, charging port had durability issues	Manufacturer sent replacement parts for devices under warranty and strengthened future device board connections
Suction Pump	Overflow valve often lost during device cleaning	Instead of replacing entire suction bottle, lid alone can be stocked as a replacement part
Radiant Warmer	Alarm batteries completely discharged during long-term storage	Manufacturer advised to pack alarm batteries separately and install alarm batteries during device installation
Temperature Monitor (Continuous)	None in field due to no qualified technology available at time of publishing	
Conductive Warmer	None in field due to no qualified technology available at time of publishing	
Respiratory Rate/Apnoea Monitor	None in field due to no qualified technology available at time of publishing	

*Abbreviations*: *CPAP* Continuous positive airway pressure, *SpO2* Oxygen saturation

## Discussion

We developed a rigorous, eight-step process to identify medical devices that are effective, affordable, rugged, and easy to use in low-resource settings and followed this process to select and implement a bundle of technologies in 65 newborn wards across Kenya, Malawi, Nigeria, and Tanzania. Of the 271 devices considered, only 45 (17%) met the TPPs based on desk research. Fourteen of 37 devices (40%) failed usability testing from evaluations with 127 clinicians. Thirty-four products passed technical laboratory evaluations and then underwent short-term environmental testing, where only one device (3%) failed. Twenty-three devices passed all evaluations, and 2197 devices were installed and continue to undergo device monitoring and quality improvement across 65 newborn wards in Kenya, Malawi, Nigeria and Tanzania.

Our findings emphasised that continued investment in research and development, commercialisation, and regulatory approval is urgently needed for devices to meet TPPs. We subjected hundreds of devices to the same rigorous evaluation process; however, only a small number (less than 30) met all performance metrics. In three of the 14 device categories recommended by the WHO as necessary for newborn care (continuous temperature monitor, conductive warmer, respiratory rate/apnoea monitor), there were no commercially available products that met TPPs. While many devices in these categories are currently under development, most remain years away from the necessary regulatory approvals for safe use in hospitals. Finding ways to accelerate the development cycle timeline while meeting all internationally recognised standards without sacrificing safety is critical.

The study revealed interesting trends in common reasons devices failed to meet TPPs, which included high initial and recurring costs, difficulty replacing or sourcing consumables, lack of appropriate regulatory approvals, and that devices were difficult for nurses to use effectively. Medical device manufacturers have an opportunity to improve current solutions or develop new devices to address these gaps. Manufacturers interested in low-resource markets could save research and development time by assessing usability of physical prototypes by a wide range of stakeholders [42].

Nearly half (40%) of evaluated devices did not pass usability testing. This finding highlights the critical need for device manufacturers to invest in user centred design (UCD). Poor usability can lead to deadly errors, longer task times, and system use avoidance. Unfortunately, usability in medical device design remains underdeveloped, with most countries engaged in design evaluations from high-resource settings [43]. Focusing usability research on high-resource countries can fail to consider the types of device errors that pose challenges in LMICs, such as critical understaffing and frequent nurse rotation between wards. WHO estimates a projected shortfall of 10 million health workers by 2030, mostly in LMIC [44] further increasing the importance and need for easy-to-use medical devices with the growth of task-shifting amidst limited human resources [45]. Therefore, to use devices quickly and effectively, implementation of user centred design for medical devices will remain essential.

One important step in ensuring the usability of medical devices in low-resource settings is to conduct significant portions of usability evaluations in the settings in which the technology will be deployed [46]. Although there may be cost and time advantages to conducting preliminary testing where the device is being designed [47], the advantages of testing in environments where the device will be used are significant. More importantly, the testing methodologies that are widely used in usability must themselves undergo a cross-cultural evaluation to determine how to best modify them to be effective in low-resource settings. This means understanding how researchers interact with users, what measures they use, and understanding cultural norms users employ when participating [46, 48]. One usability tool that is likely stable across cultures and socio-economic factors is the heuristic evaluation. Fundamental heuristics represent basic elements of product use, which do not drastically change by setting.

In user centred design, similar considerations also need to be made. Understanding how to better employ ethnographic methods effectively [49], how to use prototypes appropriately [50], how to appropriately incorporate gender, education, and cultural issues into user selection [51], simplifying UCD practices where appropriate [52], and understanding how to collect the right contextual information [53] are all important elements in ensuring that UCD is effectively used in low-resource settings.

Almost all evaluated devices passed short-term environmental testing. This unexpected result leads us to believe longer-term environmental exposure under actual, clinical-use conditions is required to surface potential failures. Furthermore, learnings from usability evaluations with nurses led us to believe that harsh environmental conditions should be evaluated in combination with the impacts of high turnover of users. Device failures seen during field evaluations were often the results of environmental factors combined with user errors (misplaced accessories, repeated device misuse causing physical damage, infrequent device cleaning and preventive maintenance, inappropriate device or consumable storage, incorrect electricity, inadequate access to spare parts or battery charging) and, in some instances, they were related to manufacturing (low durability of component parts). We predict accounting for high user turnover during short-term or long-term environmental testing would produce a higher rate of device failure.

Supporting the entire ecosystem surrounding device implementation is critically important. Procurement of devices can be difficult if local distributors do not support a particular geography; therefore, we created a distributor network that supports device installation, device monitoring, and clinical and technical trainings on proper device use and maintenance. It is critical to ensure devices are on national procurement lists to ensure they get into hospitals and additionally essential to strengthen the supply chain to ensure provision of required consumables and spare parts. Our team implemented device monitoring as well as quality improvement processes to understand device and user failures that may lead to poor device uptake during implementation.

### Strengths and limitations

Strength of our approach is that we subjected a large number of medical devices to the same rigorous evaluation process, using consensus-driven TPPs as a benchmark for performance. Limitations of our process include usability evaluations were conducted with a representative sample of clinicians from Malawi, which may not capture user needs in all low-resource settings. Nevertheless, selecting and designing medical devices specifically for the constraints of low-resource settings can help to prevent many unnecessary deaths.

## Conclusions

An evidence-based device selection process can help improve procurement of effective, affordable, rugged, and usable newborn care devices for low-resource hospitals, and feedback to manufacturers can improve device quality. Results have been shared with national and global procurement agencies to ensure qualified technologies for hospital-based care of small and sick newborns are on national procurement lists. A similar process could be adapted for use beyond newborn care to identify medical devices suitable for implementation in any low-resource setting.

## Supplementary Information

**Additional file 1.** Technical testing protocols. Technical testing protocols by product category.

**Additional file 2.** Environmental testing protocols. Environmental testing protocols for heat, humidity, dust, and power, performance functionality protocol by product category.

**Additional file 3.** Heuristic methods protocol. Protocol for heuristic and cognitive walkthroughs.

**Additional file 4.** Usability methods protocol. Protocol for usability evaluations with human subjects (end users).

**Additional file 5.** Product Category Report Cards. Report cards by product category with device make and model anonymised.

## Data Availability

All data generated or analysed during this study are included in the article or uploaded as supplementary information.

## Abbreviations

AC: Alternating Current
CPAP: Continuous Positive Airway Pressure
ENAP: Every Newborn Action Plan
IEC: International Electrotechnical Commission
ISO: International Organization for Standardization
LMIC: low- and middle-income countries
LPM: Litres per minute
mL: Millilitre
NEST360: Newborn Essential Solutions and Technologies
SDG: Sustainable Development Goal
SpO2: Oxygen saturation
SSNC: Small and sick newborn care
SUS: System Usability Survey
TPP: Target Product Profile
UCD: User centred design
WHO: World Health Organization
