# Supplementary material for: Avoid equipment graveyards: rigorous process to improve identification and procurement of effective, affordable, and usable newborn devices in low-resource hospital settings
Source: BMC Pediatr. 2023 Nov 15;23(Suppl 2):569. doi: 10.1186/s12887-023-04362-x (PMC10652423; doi:10.1186/s12887-023-04362-x)
Supplement: Supplementary file 1 — Additional file 1. Technical testing protocols. Technical testing protocols by product category. [file 12887_2023_4362_MOESM1_ESM.pdf]

Technology Testing Protocols Overview

The purpose of the development of these protocols was to characterize laboratory-based performance of each device as an input to the Product Category Report Cards.

These test protocols were developed based on the performance and operational characteristics defined in the Target Product Profiles (TPPs), hosted by UNICEF.

Each characteristic of the TPPs is documented in the relevant protocol. The values of these characteristics were determined using the documentation provided by the manufacturer with each device as well as measuring performance with standardized test equipment. Where applicable, test methods were developed in accordance with relevant IEC or ISO standard for medical electrical equipment.

Each laboratory test protocol outlines the test equipment, procedures, and metrics used to quantify each requirement of the TPP. Each device was tested three times, each with a different tester. Two devices of each candidate technology were evaluated to account for any manufacturing defects.

Contents

Technology Testing Protocols Overview 1
Syringe Pump 2
Bilirubinometer 4
Phototherapy 6
Glucometer 9
Hemoglobinometer 12
Bubble CPAP 13
Flow Splitter 15
Oxygen Concentrator 17
Pulse Oximeter (Continuous) 23
Suction Pump 26
Radiant Warmer 28

Syringe Pump

Candidate technology	
**Device make/model**	
**Device SN**	
**Test Date**	
**Tester name**	
**Tester initials & date (once complete)**	

Required tools:

- Scale - calibrate scale (if required)
- Syringes (various brands, 5-60 mL)
- Infusion line
- Beaker (to collect infusion)
- Water

☐ **Benchtop Measurement Accuracy (flow rate)**

1. Weigh syringe (before) and collection beaker (after) to confirm how much water was delivered

Syringe size & flow rate: ________________________________**Accuracy (+/-%):** ______________

Syringe size & flow rate: ________________________________**Accuracy (+/-%):** ______________

Syringe size & flow rate: ________________________________**Accuracy (+/-%):** ______________

☐ **Flow rate** **Minimum flow rate allowed:** ______________

Maximum flow rate allowed: ______________

☐ **Occlusion detection settings**

____

____

Syringe requirements

What sizes & brands does device accept? Can device program/calibrate additional brands?

____

____

☐ **Drug Library** **Yes/No:** ______________

☐ **Alarm characteristics**

____

____

☐ **Size (observed)**

____

☐ **Weight (without batteries)** **Measured weight** ______________

☐ **Power source (AC and/or battery)**

____

☐ **Battery (rechargeable? Expected length under use?)**

____

☐ **Voltage**

____

☐ **Decontamination**

____

Bilirubinometer

Candidate technology	
**Device make/model**	
**Device SN**	
**Test Date**	
**Tester name**	
**Tester initials & date (once complete)**	

Accuracy, Linearity, and Precision

Testing will be conducted using adult whole blood, adjusted to a representative neonatal hematocrit, spiked with bilirubin. For linearity and comparison assessment, we will measure 7 levels of bilirubin spanning the clinical range (approximately 0 to 30 mg/dL), with 5 replicates each on candidate technologies and a gold standard reader (UNISTAT). The studies will be performed on two devices, with one lot of strips, on a single day.

Date Testing Started:			Date Testing Finished:		
Researcher(s):					
Stock Bilirubin product #:			Bilirubin Lot number:		
Date bilirubin opened:		Date bilirubin expires:		Date stock prepared:	
Date blood drawn:			Anticoagulant:		
HCT measured via:			HCT of original donor blood (%)		

Table 1: Bilirubin dilution volumes

Level	Low bili (uL)	High bili (uL)
1	2160.0	0.0
2	1800.0	360.0
3	1440.0	720.0
4	1080.0	1080.0
5	720.0	1440.0
6	360.0	1800.0
7	0.0	2160.0

Level	Low bili (uL)	High bili (uL)
1	1800.0	0.0
2	1500.0	300.0
3	1200.0	600.0
4	900.0	900.0
5	600.0	1200.0
6	300.0	1500.0
7	0.0	1800.0

Procedure:

1. Remove plasma from participant blood and measure HCT with machine.
   a. HCT machine calculation of study blood: _______ %
2. Make stock high bilirubin & low bilirubin whole blood samples at target hematocrit (50% ±2 HCT). Make __ mL of each.
3. Make 7 dilutions using *X* uL of the “Low” stock and *Y* uL of the “High” stock, following Table 1 (circle the table used).
4. Measure hematocrit on each dilution
5. Perform user calibration on device.
6. Do replicates 1-5 on device for all 7 levels.
7. Do replicates 1-5 on device for all 7 levels.
8. Spin down each level and measure on UNISTAT.
9. Clean/disinfect entire workspace and put away/throw away materials used.

Phototherapy

This test protocol was developed based on the criteria listed in the TPPs. Where applicable, measurements were made as described in IEC 60601-2-50:2009 – Medical electrical equipment - Part 2-50: Particular requirements for the basic safety and essential performance of infant phototherapy equipment.

Candidate technology	
**Device make/model**	
**Device SN**	
**Test Date**	
**Tester name**	
**Tester initials & date (once complete)**	

Required tools:

- Watt meter
- Spectrometer (400-550 nm range) and measurement software
  Device make/model: ____
- Measuring tape
- Printed grid with boxes 10cm x 10cm (as specified by IEC 60601-2-50)

Setup instructions: **Complete** ☐

- Device must be pre-aged for 5 hours *or* length of time detailed in manual
- Spectrometer must be calibrated as described in the manual

Bulb lifetime: **Reported (hours):** _______________

Record bulb lifetime listed in manual.

Effective treatment area **Reported (cm^2):** _______________

Record bulb lifetime listed in manual.

Center the phototherapy light over the measurement area. Set height to optimal height as detailed in manual. If a range of heights is given, set height at the center of the range.

Record height of device: Height (cm): _______________

Divide the measuring area into rectangular surfaces as described in **Figure 1.** The grid should cover the entire irradiated area. The grid will divide the area into *m* x *n* rectangles such that distance between the centers of the rectangles should not exceed 10 cm.

Rectangle centers will be numbered starting at A-1 in the far-left corner. Points will proceed to A-2, A-3, etc. from left to right, and A-1, B-1, etc. from back to front.

Take measurements at the centers of the rectangles using the spectrometer in both *standard* and *intensive* modes.

Total irradiance for bilirubin (E_{bi}) at one measurement point is the result of the numerical integration of values between wavelengths 400-550 nm.

Record the number of points left-to-right (N) in which irradiance is > 10 µW/cm^2/nm N:___________

The measured area is N x 100 cm^2: **Measured (cm^2):** ______________

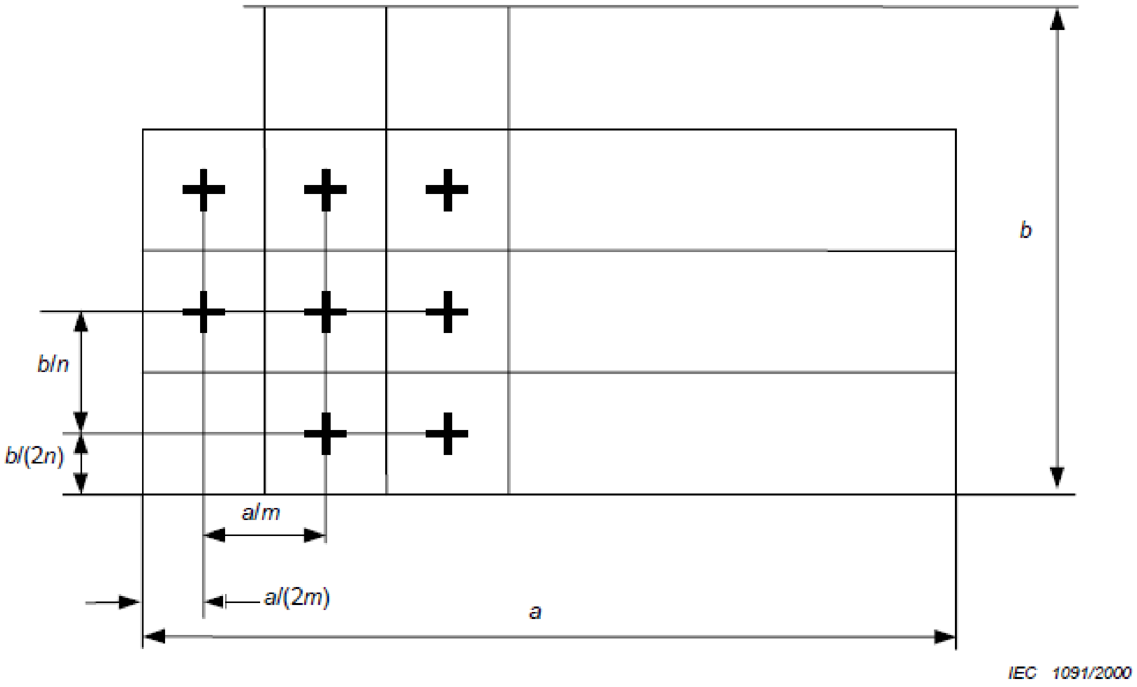

Figure 1: Measurement grid

Irradiance

Make measurements as above in both *standard* and *intensive* mode. Report average measurement across measurement area (only including points included in measurement area reported above):

Standard (uW/cm^2/nm): ____________

Intensive (uW/cm^2/nm): ____________

The ratio of the minimum irradiance measurement E_{bi} to the maximum irradiance E_{bi} should be >40%:

Minimum E_{bi}: _____________

Maximum E_{bi}: _____________

Ratio $E_{bi\ min}/E_{bi\ max}$: _____________

Light source **Observed (type):** _____________

Record light source (LED, fluorescent, etc.) listed in manual. Ensure that reported light source is correct.

Peak wavelength **Reported peak (nm):** Standard: _____

Reported peak (nm): Intensive: _____

Record peak wavelength provided in manual. If only one is provided, record it in both “standard” and “intensive” spaces.

Using data from measurements as above, identify frequency with highest intensity. Average highest intensity frequency across measurement area.

Measured peak (nm): Standard: _____

Measured peak (nm): Intensive: _____

Ease of replacing bulbs

Attempt to access and replace bulbs as described in the manual. If there is no procedure in the manual, mark N/A. Record any specialized tools required for bulb changes. Record any difficulty replacing bulbs.

____

____

Irradiance meter **Included (Y/N):** _____________

If an irradiance meter was included with the device, mark “Y”. If no irradiance meter was included, mark “N”.

Measure irradiance as described above with the meter included with the device. Record measurements and compare against measurements made with spectrometer (irradiance meter – spectrometer).

Average error: _____________

Voltage **Voltage/freq. (V/Hz):** ____________

Record the voltage and frequency listed near mains power input.

Susceptibility to power surges and sags See environmental test protocol for full procedure.

Power consumption **Reported power (W):** _____________

Record operating power recorded in manual.

Measure wattage by plugging device into watt meter and watt meter into mains power.

Power ($uW/cm^2/nm$): Standard: _____

Power ($uW/cm^2/nm$): Intensive: _____

Glucometer

Candidate technology	
**Device make/model**	
**Device SN**	
**Test Date**	
**Tester name**	
**Tester initials & date (once complete)**	

Required tools:

- Record lot # of test strips: ____________________
- Standard Reference Material 917c D-Glucose (Dextrose)
- Flask for preparing standard glucose solution

Setup instructions:

Use Standard Reference Material 917c D-Glucose (Dextrose) ID# ______________________________

Accuracy & Precision

Prepare Standard Reference Material 917c D-Glucose (Dextrose) as described in reference materials instructions to a mixture of **45 mg/dL**

Measure and report the result of 3 new test strips:

Test strip #1 (mg/dL): ________________

Test strip #2 (mg/dL): ________________

Test strip #3 (mg/dL): ________________

Calculate average and range of 3 samples:

Average (mg/dL): ___________________

Range (mg/dL): ___________________

Report difference from true (**45 mg/dL)**:

Test strip #1 (+/-mg/dL): __________

Test strip #2 (+/-mg/dL): __________

Test strip #3 (+/-mg/dL): __________

Prepare Standard Reference Material 917c D-Glucose (Dextrose) as described in reference materials instructions to a mixture of **54 mg/dL**

Measure and report the result of 3 new test strips:

Test strip #1 (mg/dL): _______________

Test strip #2 (mg/dL): _______________

Test strip #3 (mg/dL): _______________

Calculate average and range of 3 samples:

Average (mg/dL): __________________

Range (mg/dL): __________________

Report difference from true (**54 mg/dL)**:

Test strip #1 (+/-mg/dL): __________

Test strip #2 (+/-mg/dL): __________

Test strip #3 (+/-mg/dL): __________

Linear range

Prepare Standard Reference Material 917c D-Glucose (Dextrose) to a mixture of **0 mg/dL**

Measure and report the result of 1 test strip

Test strip #1 (+/-mg/dL): __________

Prepare Standard Reference Material 917c D-Glucose (Dextrose) to a mixture of **900 mg/dL**

Measure and report the result of 1 test strip

Test strip #1 (+/-mg/dL): __________

Display **Qual./Quant:** __________

Record whether reported results are qualitative or quantitative.

Sample specimen

Record the quantity of blood listed in the manual that is required for accurate reading. Record whether the device takes whole blood.

____

Calibration

Record calibration method listed in manual. If no method is listed, write “N/A”.

____

Footprint

Record the size and weight of the device listed in the manual. Record whether any consumables (i.e. batteries) are listed in the manual.

____

Kit stability and storage conditions

Record any storage requirements listed in the manual.

____

Voltage **Voltage/freq. (V/Hz):** ______________

Record the voltage and frequency listed near the power cord.

Battery powered

Record how long the device is expected to operate on a single charge during continuous use.

____

Hemoglobinometer

Candidate technology	
**Device make/model**	
**Device SN**	
**Test Date**	
**Tester name**	
**Tester initials & date (once complete)**	

% of RBC-containing stock	Approximate Hb (g/dL)	uL RBC-containing stock	uL plasma
**100**	20	650.0	0
**85**	17	552.5	97.5
**70**	14	455.0	195.0
**55**	11	357.5	292.5
**40**	8	260.0	390.0
**25***	5	162.5	487.5
**10**	2	65.0	585.0
**0**	0	0	650.0

Accuracy and Linear Range

- Sample preparation
  - Collect venous blood in EDTA tubes. You need 2 tubes of 6 mL each per day.
  - Make the hemoglobins dilutions from 0-20 g/dL, and then use a second tube to get 20-25 g/dL. The higher hemoglobin concentrations are viscous and harder to work with. These parts can be done in either order.
- Testing procedure
  - Mix well before EVERY MEASUREMENT. Red blood cells begin to settle very quickly when the tube sets on the bench, and even 2 minutes between mixing and sampling can affect your results.

Procedure:

1. Collect venous blood in EDTA tubes. You need 2 tubes of 6 mL each per day.
2. Make the hemoglobins dilutions from 0-20 g/dL, and then use a second tube to get 20-25 g/dL. The higher hemoglobin concentrations are viscous and harder to work with. These parts can be done in either order.
3. Measure hemoglobin on each dilution
4. Perform user calibration on device.
5. Do replicates 1-5 on device for all 7 levels.
6. Do replicates 1-5 on device for all 7 levels.
7. Spin down each level and measure on Act Diff.
8. Clean/disinfect entire workspace and put away/throw away materials used.

Bubble CPAP

Candidate technology	
**Device make/model**	
**Device SN**	
**Test Date**	
**Tester name**	
**Tester initials & date (once complete)**	

Required tools:

- O2 Concentrator
  - Record make/model/SN: ______________________________
- Gas flow analyzer OR pressure meter and flow meter
  - Record make/model/SN: ______________________________

Flow Driver **Y/N:** ______________

Record whether the device has an on-board air compressor.

Oxygen Flow Capability

Record the minimum O2 flow reported in the manual. **Min. O_2 (L/min):** _____

Record the minimum O2 flow reported in the manual. **Max. O_2 (L/min):** _____

Pressure

Using a gas flow analyzer, measure the MINIMUM pressure provided by the device, with the straw submerged to 4 cm H_2O and flow set to 4 L/min.

Min. press. (cm H_2O): ______________

Using a gas flow analyzer, measure the MAXIMUM pressure provided by the device, with the straw submerged to 8 cm H_2O and flow set to the highest possible setting.

Max. press. (cm H_2O): ______________

Total (blended) Flow

Using a gas flow analyzer, measure the MINIMUM (non-zero) total (blended) flow.

Min. flow (L/min): ______________

Using a gas flow analyzer, measure the MAXIMUM total (blended) flow.

Max. flow (L/min): ______________

Humidification

Record whether the device provides any method of humidification.

____

Alarms

Cut power to the device without first turning the device off. Record whether an alarm occurs.

____

Reduce flow below specified flow rate (e.g. by causing a leak in tubing). Record whether an alarm occurs.

____

Reduce pressure below specified pressure (e.g. by causing a leak in tubing). Record whether an alarm occurs.

____

Consumables **Necessary (Y/N):** _______________

Record whether any consumables (i.e. tubing) are listed in the manual, or if you believe one would be required to perform any task listed in the manual. Cleaning supplies do not count as consumables for the purpose of this question.

Accessories

Record whether any accessories (i.e. tubing) are proprietary or non-proprietary.

____

Back-up Battery

Cut power to the device without first turning the device off. Record whether the device continues to function. Record battery life.

____

Voltage **Voltage/freq. (V/Hz):** _______________

Record the voltage and frequency listed near the power cord.

User instructions **Included (Y/N):** _______________

Record whether user instructions shipped with the device.

Flow Splitter

Candidate technology	
**Device make/model**	
**Device SN**	
**Test Date**	
**Tester name**	
**Tester initials & date (once complete)**	

Required tools:

- Flow meter
- Oxygen concentrator capable of delivering 10 L/min of flow
  - Record make/model/SN: ______________________________
- Flow meter
  - Record make/model/SN: ______________________________

Air flow per patient **Observed:** _______________

Record the flow/patient listed in the manual.

Using a flow meter, measure the maximum air flow from each outlet.

Measured (L/min): **Outlet 1:** ______

Outlet 2: ______

Outlet 3: ______

Outlet 4: ______

Outlet 5: ______

Flow control **Pass/Fail:** _______________

Connect flow splitter to flow driver.

Open each patient outlet to maximum flow one at a time. Ensure that all other patient outputs remain at 0 L/min.

Open one patient outlet to maximum flow. Open a second outlet and check whether flow rate on the first outlet changes (ensure that flow provided by driver is sufficient for both outlets). If change in other outlets is < 20%, mark "PASS". Otherwise, mark "FAIL".

Number of outputs **Observed:** _______________

Record the number of outlets on the splitter.

Visual indicators for flow rate **Yes/no:** _______________

Record whether there are visual indicators for flow rate.

Markings (L/min) **L/min:** _______________

Record the incremental markings on the flow meters (i.e. ¼ L/min, ½ L./min)

Oxygen Concentrator

This test protocol was developed based on the criteria provided in the TPPs. Where applicable, measurements were made as described in ISO 80601-2-69:2014 – Medical electrical equipment - Part 2-69: Particular requirements for basic safety and essential performance of oxygen concentrator equipment.

Candidate technology	
**Device make/model**	
**Device SN**	
**Test Date**	
**Tester name**	
**Tester initials & date (once complete)**	

Required tools:

- UltraMaxO2 oxygen analyzer OR alternative flow/pressure/oxygen meter
- 100% O2 source (for calibration of oxygen meter)
- Sound level meter
- Sound level meter calibrator
- Tripod
- Tape measure
- Watt meter
- Luggage scale
- Stopwatch

Required setup:

- Calibrate sound meter as described in sound meter user manual
- Calibrate flow/pressure/oxygen meter as described in flow/pressure/oxygen meter user manual

Flow meter **Observed #:** ______________

Record the number of flow meters on the device.

Flow rate **Observed range (L/min):** _____________

Obs. resolution (L/min): _____________

Record the range and resolution written on the flow meter(s).

Open the flow meter(s) to the maximum marked flow rate. Measure the flow rate using the flow meter.

Meas. flow (L/min): Outlet 1: ________

Outlet 2: ________

Time to reach 95% of spec. performance **Measured (mm:ss):** ______________

Turn on device with oxygen meter attached. Record the amount of time it takes to reach 95% of the oxygen concentration specified in the manual.

Record specified O2 output (%) From manual (%): _____________

Oxygen purity **Measured (%): _____________**

Turn on device with oxygen meter attached. Record final oxygen concentration once concentration has reached steady state.

Audible alarms **For high temp. (Y/N): _____________**

Record whether the manual lists an alarm state for high temperature.

For low flow rate (Y/N): ____________

Record whether the manual lists an alarm state for low flow rate.

For high/low press. (Y/N): __________

Generate a high pressure (e.g. by occluding tubing). Record whether a pressure alarm exists.

Indicators **Y/N: _____________**

Are indicators for lights and buttons clearly marked?

Record any concerns regarding indicator clarity below:

____

Mobility **Four casters (Y/N): ______________**

Two brakes (Y/N): ______________

Record whether the device has casters. If yes, record whether there are at least 2 brakes.

Oxygen monitor **Aud./vis. status (Y/N): _____________**

Color coding (Y/N): ______________

Record whether there is an oxygen monitor that indicates the percentage of oxygen provided by the device. If yes, record if it provides both visual and auditory information. Record whether the visual information provided is color coded.

Oxygen Outlet **Observed #: ______________**

Record the number of oxygen outlets.

Recessed (Y/N): ______________

Record whether those oxygen outlets are recessed (protected).

Metal (Y/N): ______________

Record if the oxygen outlets are metal.

Sound level

Calibrate sound level meter using calibrator and calibration instructions with device. Set settings to: A frequency mode, SLOW integration, RANGE: 30-90dB.

Use tripod to set sound level meter 1m from 0.15cm above the geometric center of device and level with the device, with microphone facing device (**position 1,** as shown in **Figure 1**).

Record ambient noise sound level: Measured (dB): _____________

Turn on device at 3LPM and wait 2 minutes for start-up. Record sound level with device on (point 1). Use tripod to set sound level meter at **positions 2-10** as described in **Table 1** and shown in **Figure 1**, below:

Table 1. Table of microphone array positions (IEC 80601-2-69)

Position number	*x/r*	*y/r*	*z/r*
1	– 0,99	0	0,15
2	0,50	– 0,86	0,15
3	0,50	0,86	0,15
4	– 0,45	0,77	0,45
5	– 0,45	– 0,77	0,45
6	0,89	0	0,45
7	0,33	0,57	0,75
8	– 0,66	0	0,75
9	0,33	– 0,57	0,75
10	0	0	1,00
11	0,99	0	0,15
12	– 0,50	0,86	0,15
13	– 0,50	– 0,86	0,15
14	0,45	– 0,77	0,45
15	0,45	0,77	0,45
16	– 0,89	0	0,45
17	– 0,33	– 0,57	0,75
18	0,66	0	0,75
19	– 0,33	0,57	0,75
20	0	0	1,00

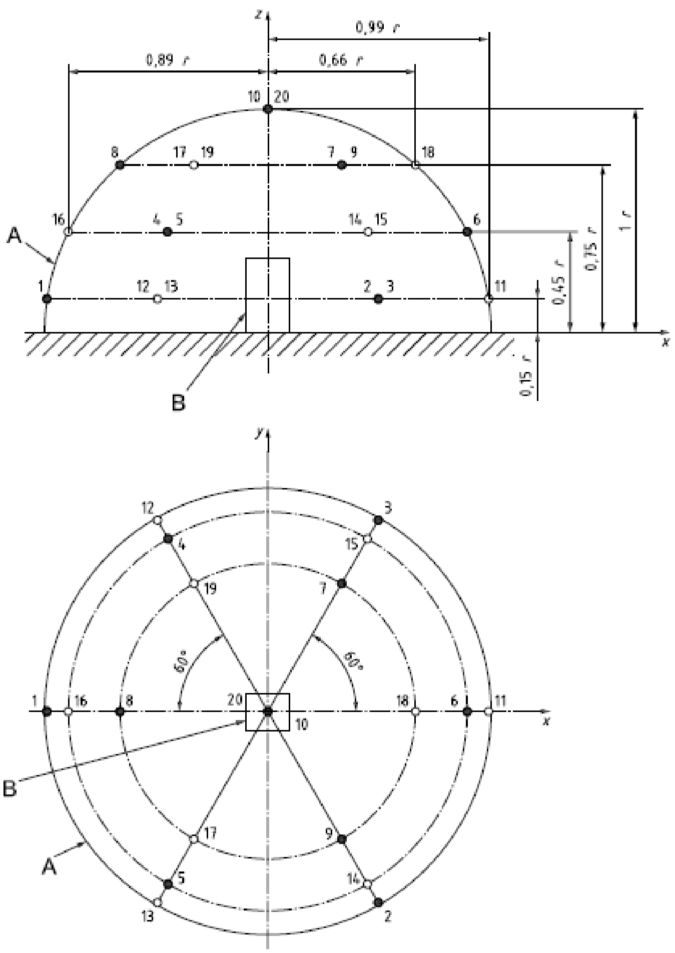

Figure 2. Diagram of microphone array positions (IEC 80601-2-69)

Measured (dB): Point 1: ____________

Point 2: ____________

Point 3: ____________

Point 4: ____________

Point 5: ____________

Point 6: ____________

Point 7: ____________

Point 8: ____________

Point 9: ____________

Point 10:____________

User instructions **Included (Y/N):** _____________

Record whether user instructions shipped with the device. If yes, record if instructions are additionally included on the device.

On device (Y/N): _____________

Weight **Measured (kg):** _____________

Using the luggage scale, measure the weight of the device.

Decontamination **Possible (Y/N):** _____________

Record any concerns regarding decontamination below:

____

____

Durability and robustness **Pass/Fail:** _____________

Record any concerns about durability and robustness below. Refer to environmental test protocol for additional details.

____

____

Filter cleaning **Interval (wks):** _____________

Record the filter cleaning interval as provided in the manual.

Hour meter **Y/N:** _____________

Record whether the device has an hour meter.

Preventive maintenance interval **Interval (months):** _____________

Record the preventative maintenance interval provided in the manual.

Replacement parts and consumables **Required (Y/N):** _____________

List replacement parts and consumables below.

____

Warranty **Observed (yrs):** _____________

Record the length of the warranty.

Technical skills required for maintenance **High/Low:** _____________

Record any concerns regarding maintenance below.

____

Tools required **Number of tools:** _____________

____

____

User care skill level **Recommended:** _____________

Refer to usability testing results.

Electrical plug **Type:** _____________

Record the plug type.

Power **Power (W):** _____________

Plug concentrator into watt meter. Turn the concentrator to the maximum flow rate specified in the user manual. Record power consumption (W).

Power efficiency **Power req./L/min (W):** _____________

Power efficiency can be calculated as power consumption (W) divided by the flow rate used above.

Power consumption

Plug concentrator into watt meter. Record power consumption (W) at 3, 5, 8, and 10 LPM.

Power at 3 L/min (W): _____________

Power at 5 L/min (W): _____________

Power at 8 L/min (W): _____________

Power at 10 L/min (W): _____________

Integrated surge protection **Y/N:** _____________

Record if integrated surge protection is included with the device.

Pulse Oximeter (Continuous)

This test protocol was developed based on the criteria provided in the TPPs. Where applicable, measurements were made as described in ISO 80601-2-61:2017 – Medical electrical equipment - Part 2-61: Particular requirements for the basic safety and essential performance of pulse oximeter equipment.

Candidate technology	
**Device make/model**	
**Device SN**	
**Test Date**	
**Tester name**	
**Tester initials & date (once complete)**	

Required tools:

- Patient simulator
  - Record make/model/SN: ______________________________
- SpO2 finger simulator
  - Record make/model/SN: ______________________________
- Scale
  - Record make/model/SN: ______________________________

Setup instructions:

Patient simulator with SpO2 finger simulator must be calibrated as described in the manual:

Pulse rate

Select appropriate R-curve for specific pulse ox (as described in patient simulator manual). Program patient simulator with finger attachment to output the reported pulse rate range.

Pulse rate range (bpm): _____________

Record the pulse rate range provided in the manual.

Set the patient simulator at the minimum pulse rate in the manual. Record the set pulse rate and measured pulse rate.

Set pulse rate (bpm): _____________

Meas. pulse rate (bpm): _____________

Set the patient simulator at the maximum pulse rate provided in the manual. Record the set pulse rate and measured pulse rate.

Set pulse rate (bpm): _____________

Meas. pulse rate (bpm): _____________

Pulse rate accuracy **Pulse rate accuracy (bpm): __________**

Record the pulse rate accuracy provided in the manual.

Set the patient simulator at the minimum pulse rate provided in the manual. Record three measurements of pulse rate and record the average difference from the set pulse rate.

Pulse rate diff. (bpm): _____________

Set the patient simulator at the maximum pulse rate provided in the manual. Record three measurements of pulse rate and record the average difference from the set pulse rate.

Pulse rate diff. (bpm): _____________

Set one odd and one even pulse rate (i.e. 63 and 64). Record pulse rate resolution.

Pulse rate resolution (bpm): _________

SpO_2

Select appropriate R-curve for specific pulse ox (as described in patient simulator manual). Program patient simulator with finger attachment to output the reported pulse rate range.

SpO_2 range (%): ____________

Record the pulse rate range provided in the manual.

Set the patient simulator at the minimum SpO_2 provided in the manual. Record the set SpO_2 and measured SpO_2.

Set SpO_2 (%): _____________

Meas. SpO_2 (%): ____________

Set the patient simulator at the maximum SpO_2 provided in the manual. Record the set SpO_2 and measured SpO_2.

Set SpO_2 (%): ____________

Meas. SpO_2 (%): ____________

SpO_2 accuracy **SpO_2 range (%): ____________**

Set the patient simulator at the minimum SpO_2 provided in the manual. Record three measurements of pulse rate and record the average difference from the set pulse rate.

SpO_2 diff. (%): _____________

Set the patient simulator at the maximum SpO_2 provided in the manual. Record three measurements of pulse rate and record the average difference from the set pulse rate.

SpO_2 diff. (%): _____________

Alarm characteristics **Visual/auditory:** _____________

Generate an alarm (i.e. low pulse rate). Record whether the alarm is visual or auditory.

Attempt to adjust alarms using procedure described in the manual. If no procedure is provided, mark "N/A".

____

Consumables **Necessary (Y/N):** _____________

Record whether any consumables (i.e. stickers) are listed in the manual, or if you believe one would be required to perform any task detailed in the manual. Cleaning supplies do not count as consumables for the purpose of this question.

Probe lifetime **Lifetime (yrs):** _____________

Record the probe lifetime provided in the manual.

Patient interface

Record the instructions provided in the manual for decontamination of the probe between patients. If no procedure is described, mark "N/A".

____

Neonatal probe available **Available (Y/N):** _____________

Record whether a neonatal probe is available for the device.

Battery **Available (Y/N):** _____________

Record whether the device is battery powered. If yes, record how long the battery lasts on a single charge. Record the voltage and frequency of mains power required for charging.

Battery life (hrs): _____________

Voltage/freq. (V/Hz): _____________

Weight **Weight (g):** _____________

Weigh device (including probe).

Size

Record portability and footprint of device (portable, not portable). Record whether the device includes a charging dock.

____

Usage Meter **Available (Y/N):** _____________

Attempt to access usage meter using procedure provided in the manual. If no procedure exists, mark "N/A".

Suction Pump

Candidate technology	
**Device make/model**	
**Device SN**	
**Test Date**	
**Tester name**	
**Tester initials & date (once complete)**	

Required tools:

- Negative pressure gauge (range -30in Hg - 0inHg)
- Sound level meter
- Sound level meter calibrator

Required setup:

- Calibrate sound level meter as described in sound level meter manual.

Pressure **Spec. min. (inHg):** ______________

Spec. max. (inHg): ______________

Range (cont./disc.): ______________

Record the specified minimum and maximum pressures as described in the manual. Record whether the range of pressures available is continuous or discrete (few, pre-specified pressures).

Set the suction pressure to the minimum (non-zero) value specified by the manual. Measure suction pressure using negative pressure gauge.

Measured min. (inHg): ______________

Set the suction pressure to the maximum value specified by the manual. Measure suction pressure using negative pressure gauge.

Measured max. (inHg): ______________

Bottle capacity **Volume (L):** ______________

Record the bottle capacity in L.

Mechanism to prevent liquids from reaching pump **Pass/Fail:** ______________

Fill bottle to capacity with water. Continue to draw liquid into bottle. Observe whether a mechanism exists to direct overflow away from pump. If yes, mark “PASS”. If no, mark “FAIL”.

Sound level

Calibrate sound level meter using calibrator and calibration instructions with device. Set settings to: A frequency mode, SLOW integration, RANGE: 30-90dB.

Use tripod to set sound level meter 1m from geographic center of device in the horizontal plane.

Record ambient noise sound level: Measured (dB): _____________

Set the suction pressure to the minimum (non-zero) value specified by the manual. Measure sound level.

Min. pres. (dB): ____________

Set the suction pressure to the maximum value specified by the manual. Measure sound level.

Max. pres. (dB): _____________

Number of steps to use **Observed:** _____________

Count the number of steps required to set up and use the device.

Collection vessel easy to clean

Record any concerns about disinfection of collection vessel on lines below:

____

____

Voltage **Voltage/freq. (V/Hz):** _____________

Record the voltage and frequency provided near the power cord.

Radiant Warmer

This test protocol was developed based on the criteria provided in the TPPs. Where applicable, measurements were made as described in IEC 60601-2-21:2009 – Medical electrical equipment - Part 2-21: particular requirements for the basic safety and essential performance of infant radiant warmers.

**Candidate technology**	
**Device make/model**	
**Device SN**	
**Test Date**	
**Tester name**	
**Tester initials & date (once complete)**	

Required tools:

- Water bath
- Reference thermometer
- Tape measure
- 5 uniformity test devices (aluminum discs of 100g with temperature probes enclosed, as described in IEC 60601-2-21)
- Stopwatch

Benchtop measurement accuracy:

Place the warmer probe and reference thermometer into the water bath within 3 cm of each other. Wait 3 minutes or until the temperature displayed on the reference thermometer and the device display has stabilized at 32, 36, and 38 °C.

Manually record the temperatures displayed on the reference thermometer and the device display. Record three measurements on each device at each temperature. Repeated measurements should be separated by 1 minute.

Table 1. Surface temperatures recorded from device

Water bath temperature	Reference temperature	Display temperature	Reference temperature	Display temperature	Reference temperature	Display temperature
32 °C						
36 °C						
38 °C						

Temperature stability:

Calculate the bias (average of display temperature-reference temperature) and 95% CI (1.96*standard deviation) of the above temperatures.

Bias (°C): ______________

95% CI (°C): ______________

Time to indicate accurate temperature:

Place a humimic gel surface approximately 4 mm in thickness and 4 cm in diameter onto water bath set at 50 °C, such that the lower surface of the gel is submerged in water but the upper surface of the gel is exposed to the air. Place the reference thermometer probe onto the upper surface of the gel and continuously monitor gel temperature. Allow 20 minutes for the gel temperature to stabilize. The final, stabilized temperature of the upper surface of the gel should be 37 ± 1 °C.

Place the patient side of the device probe firmly against the gel surface while simultaneously starting the stopwatch; manually record the temperature displayed on device display at the times listed in Table 2, below.

Table 2. Surface temperatures recorded from device

Time (min:sec)	Display temperature	Time (min:sec)	Display temperature	Time (min:sec)	Display temperature
0:02		0:35		2:15	
0:04		0:40		2:30	
0:06		0:45		2:45	
0:08		0:50		3:00	
0:10		0:55		3:15	
0:12		1:00		3:30	
0:14		1:05		3:45	
0:16		1:10		4:00	
0:18		1:15		4:15	
0:20		1:20		4:30	
0:22		1:25		4:45	
0:24		1:30		5:00	
0:26		1:40			
0:28		1:50			
0:30		2:00			

Record the time at which temperature stabilized (did not change by > 0.1°C):

Time (sec): ______________

Uniformity:

Place 5 test devices at the centers of quadrants as shown in Figure 1, below.

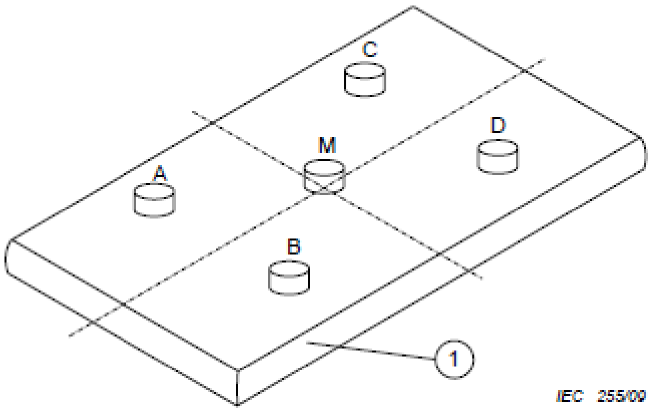

Figure 1. Uniformity test device locations (IEC 60601-2-21)

Place a temperature sensor inside each test device, and firmly affix the patient probe to the top of device "M" as described in IEC 60601-2-21. Set control temperature to 36 °C.

Record temperatures of test devices every two minutes for one hour.

Manually record temperature measurements for all five test devices and display temperature at the times in Table 3, below.

Table 3. Temperatures measured at 5 locations on radiant warmer bed.

Time (min)	"A" temp.	Time (min)	"B" temp.	Time (min)	"C" temp.	Time (min)	"D" temp.	Time (min)	"M" temp.	Time (min)	Display temp.
2		2		2		2		2		2	
4		4		4		4		4		4	
6		6		6		6		6		6	
8		8		8		8		8		8	
10		10		10		10		10		10	
12		12		12		12		12		12	
14		14		14		14		14		14	
16		16		16		16		16		16	
18		18		18		18		18		18	
20		20		20		20		20		20	
22		22		22		22		22		22	
24		24		24		24		24		24	

26		26		26		26		26		26	
28		28		28		28		28		28	
30		30		30		30		30		30	
32		32		32		32		32		32	
34		34		34		34		34		34	
36		36		36		36		36		36	
38		38		38		38		38		38	
40		40		40		40		40		40	
42		42		42		42		42		42	
44		44		44		44		44		44	
46		46		46		46		46		46	
48		48		48		48		48		48	
50		50		50		50		50		50	
52		52		52		52		52		52	
54		54		54		54		54		54	
56		56		56		56		56		56	
58		58		58		58		58		58	
60		60		60		60		60		60	

Calculate the bias (average of display temperature-reference temperature) and 95% CI (1.96*standard deviation).

Bias (°C): A: _____________

95% CI (°C): A: _____________

Bias (°C): B: _____________

95% CI (°C): B: _____________

Bias (°C): C: _____________

95% CI (°C): C: _____________

Bias (°C): D: _____________

95% CI (°C): D: _____________

Alarm characteristics **Visual/auditory:** _________________

Generate an alarm (i.e. low temperature) and record whether visual and auditory alarms exist.

Alarm limits **Adjustable (Y/N)**: _______________

Attempt to adjust alarm limits, as described in the manual. If there is no procedure in the manual, mark "NO".

Consumables **Necessary (Y/N):** _______________

Record whether any consumables (i.e. stickers) are listed in the manual, or if you believe one would be required to perform any task detailed in the manual. Cleaning supplies do not count as consumables for the purpose of this question.

Decontamination

Record any specific concerns regarding decontamination here:

____

____

APGAR timer **Included (Y/N):** _______________

Attempt to set APGAR timer as described in the manual. If there is no procedure in the manual, mark "NO".

Scale **Included (Y/N):** _______________

Attempt to use scale as described in the manual. If there is no procedure in the manual, mark "NO".

Power consumption **Total W:** _______________

Measure wattage by plugging device into watt meter and watt meter into mains power.

Mobility **Wheels (Y/N):** _______________

Unlock the wheels and attempt to move the device. If the device moves easily, mark "YES". If there are no wheels, mark "NO".

Operating temperature **Recorded (°C):** _______________

Record operating temperature provided in manual. See environmental test results for complete data set.

Patient interface **Reusable (Y/N):** _______________

Record whether any patient interface (i.e. stickers) are listed in the manual, or if you believe one would be required.

Temperature control **Auto infant temp. (Y/N):** _______________

Record whether the temperature of the device will automatically adjust in response to the infant's temperature (e.g. servo mode).

Training required **Minimal (Y/N):** _______________

Record whether the instructions in the manual appear to be sufficient to perform necessary tasks.

User instructions **Included (Y/N):** ______________

Record whether user instructions shipped with the device.

Voltage **Voltage/freq. (V/Hz):** ______________

Record the voltage and frequency provided near the power cord.
