# Supplementary material for: Avoid equipment graveyards: rigorous process to improve identification and procurement of effective, affordable, and usable newborn devices in low-resource hospital settings
Source: BMC Pediatr. 2023 Nov 15;23(Suppl 2):569. doi: 10.1186/s12887-023-04362-x (PMC10652423; doi:10.1186/s12887-023-04362-x)
Supplement: Supplementary file 2 — Additional file 2. Environmental testing protocols. Environmental testing protocols for heat, humidity, dust, and power, performance functionality protocol by product category. [file 12887_2023_4362_MOESM2_ESM.pdf]

Environmental testing protocols for heat, humidity, dust, and power

The following protocols outline steps to test medical device performance after exposure to high heat, exposure to high heat with extreme humidity, exposure to large volumes of fine dust, and simulated electrical power instability and complete power failure.

Heat Test Protocol

This protocol is in accordance with IEC 60068-2-2 Be, whose paragraphs are referenced below.

Required Tools

- Candidate technology (n=1)
- Performance functionality testing data sheet
- Environmental chamber with specifications from ambient to 50 °C, large enough to enclose the entire test subject (6.2), and with the ability to maintain relative humidity (rH) at 50% or below (6.8.2).

Protocol

- ❑ Perform baseline performance functionality testing of candidate technology.
- ❑ Place the test subject in the chamber and turn candidate technology on.
- ❑ Close the chamber and raise the temperature to 50 °C at a rate no faster than 1 degree K per minute averaged over a period of not more than 5 minutes (5.1).
- ❑ Allow the test to run for 16 hours (6.5.2).
- ❑ Lower the temperature of the chamber to an ambient temperature at a rate no faster than 1 degree K per minute averaged over a period of not more than 5 minutes (5.1).
- ❑ Perform performance functionality testing of candidate technology.
- ❑ (1) Note any deviations from procedure (7)
- ❑ (2) Note if chamber is high or low airflow. If it is high airflow, note temperature difference.

1	Deviations in procedure	
2	High or low airflow chamber? If high, temperature difference	

Humidity (Damp Heat) Test Protocol

This protocol is in accordance with IEC 60068-2-30, severity A, variant 1, whose paragraphs are referenced below.

Required Tools

- Candidate technology (n=1)
- Performance functionality testing data sheet
- Environmental chamber with specifications from 25 °C to 40 °C with a tolerance of 3 degrees Kelvin and relative humidity (rH) capabilities up to 95% (4.1). Water used in the chamber shall have a resistivity no less than 500 ohms (4.5).

Protocol

- ❏ Perform baseline performance functionality testing of candidate technology.
- ❏ Ensure that the subject's temperature is stabilized (7.2).
- ❏ Place the test subject in the chamber and seal it appropriately. Turn the device on.
- ❏ Raise the rH to 95% with a temperature of 25 °C (7.2).
- ❏ Once the temperature and rH are stable, raise the temperature to 40 °C at a rate of 1 degree every 10 minutes. (7.3.1)
- ❏ Allow the test to run at 40 °C and 95% rH for 10 hours (7.3.2)
- ❏ Lower the temperature to 25 °C at a rate of 1 degree every 12 minutes (7.3.3)
- ❏ Lower the humidity to 75% rH, this should occur in under 10 minutes (9).
- ❏ Remove the test subject from the chamber.
- ❏ Perform performance functionality testing of candidate technology.
- ❏ (1) Note any deviations in procedure

1	Deviations in procedure	

Dust Test Protocol

This protocol is in accordance with IEC 60068-2-68 La1, category 2, whose paragraphs are referenced below.

Required Tools

- Candidate technology (n=1)
- Performance functionality testing data sheet
- Oven with capability to reach and maintain 80 °C
- 2250 grams Arizona test dust – A3 medium (4.1.4.2)
- Square-meshed sieve with a nominal wire diameter of 50 μm and a nominal width between wires of 75 μ.m. (4.1.4.1)
- Humidity sensor
- Dust test chamber (temperature and humidity controlled, contains fan to recirculate dust)

Protocol

- ❑ Bake test dust at 80 °C for 2 hours (4.1.4.1)
- ❑ Ensure all dust can pass through sieve (4.1.4.1)
- ❑ Perform baseline performance functionality testing of candidate technology.
- ❑ (1) Plug in dust test chamber and lower humidity to 20% rH. When the humidity sensor indicates rH of 20% or below, proceed to the next step. If the specified rH cannot be achieved, raise the temperature of the chamber. Do not allow the temperature of the chamber to exceed 40 °C. (4.1.4.5)
- ❑ (2) More than one test subject may be tested at once ONLY if both (4) and (6) are YES for total volume of all test subjects and total base of all test subjects. Note the names of additional test subjects if applicable (4.1.3)
- ❑ (3) Calculate total volume test subject(s).
- ❑ (4) Note if volume test subject(s) is under 25% test chamber volume
- ❑ (5) Note area of subject(s) test base
- ❑ (6) Note if area is under 50% horizontal working space
- ❑ Place all test subject(s) in dust test chamber. Ensure devices are not touching and they do not shield each other from dust. (4.1.7)
- ❑ (7) Note if the position of the device is different than normal operating position (4.1.12)
- ❑ Switch on devices.
- ❑ Turn on dust. Let test run for 4 hours (4.1.4.7)
- ❑ Turn off dust and allow dust to settle 2 hours. (4.1.9)
- ❑ Wipe down test subject (4.1.10)
- ❑ Perform performance functionality testing of candidate technology.

1	% rH achieved	

2	Name additional test subjects	
3	Volume test subject(s) (cm^3)	
4	Volume exceeds 25% test chamber volume? (y/n)	
5	Area test subject(s) base	
6	Area exceeds 50% test chamber horizontal working space y/n	
7	Position of device if irregular	

Power Surge/Sag Test Protocol

This protocol is in accordance with IEC 61000-4-14, whose paragraphs are referenced below. Three separate tests, according to Classes 2, 3, and X, will be performed. Class X is used to test complete power loss.

Required Tools

- Candidate technology (n=1)
- Performance functionality testing data sheet
- Programmable power supply, plugged into 30A wall outlet.
  - Class 2; ± 8%, sequence performed 3 times.
  - Class 3; ± 12%, sequence performed 3 times.
  - Class X; ± 100%, sequence performed 3 times.

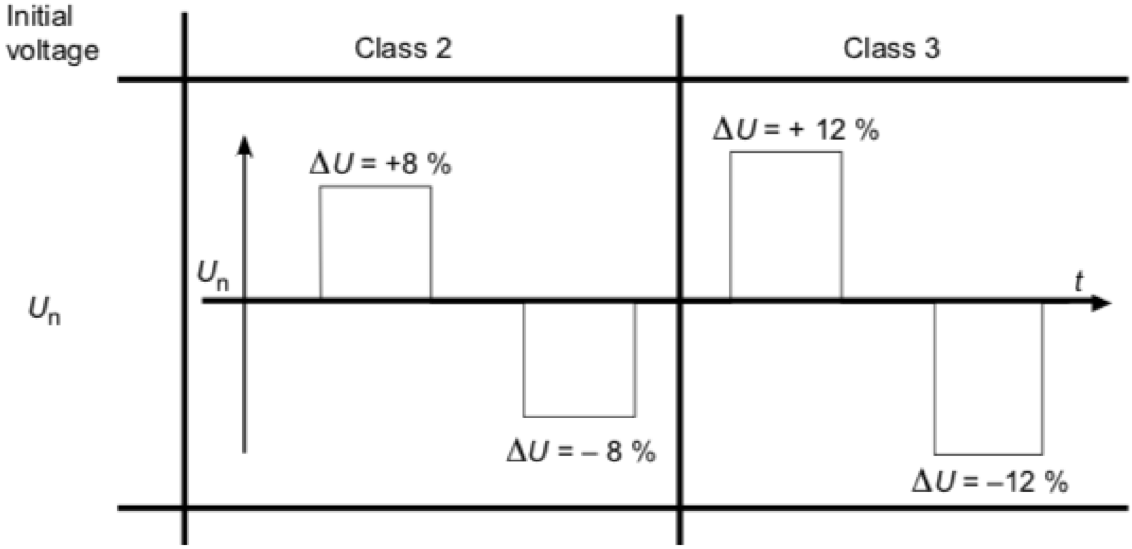

Protocol

- ❑ Perform baseline performance functionality testing of candidate technology.
- ❑ (1) Note power supply specifications (make, model, SN)
- ❑ Calibrate the programmable power supply as instructed in user manual
- ❑ (2) Note the following: information on possible connections (plugs, terminals, etc.) and corresponding cables and peripherals on test device (8)
- ❑ (3) Note input power port of device (8)
- ❑ (4) Describe test setup (8)
- ❑ Ensure the power supply is programmed in accordance with Class 2 (8.1)
- ❑ (5) Begin the test sequence. Take notes on the behavior of the device throughout.
- ❑ Ensure the power supply is programmed in accordance with Class 3 (8.1)
- ❑ (6) Begin the test sequence. Take notes on the behavior of the device throughout.
- ❑ Ensure the power supply is programmed in accordance with Class X (8.1)
- ❑ (7) Begin the test sequence. Take notes on the behavior of the device throughout.
- ❑ Perform performance functionality testing of candidate technology.

1	Power supply specifications (Make, Model, SN)	
2	Information on connections (plugs, terminals, cables)	
3	Input power port of device	
4	Test setup description	
5	Notes on behavior during Class 2	
6	Notes on behavior during Class 3	
7	Notes on behavior during Class X	
