# Supplementary material for: Avoid equipment graveyards: rigorous process to improve identification and procurement of effective, affordable, and usable newborn devices in low-resource hospital settings
Source: BMC Pediatr. 2023 Nov 15;23(Suppl 2):569. doi: 10.1186/s12887-023-04362-x (PMC10652423; doi:10.1186/s12887-023-04362-x)
Supplement: Supplementary file 3 — Additional file 3. Heuristic methods protocol. Protocol for heuristic and cognitive walkthroughs. [file 12887_2023_4362_MOESM3_ESM.pdf]

Heuristic and Cognitive Walkthrough Protocols

Heuristic results were used to eliminate candidate technologies with potentially catastrophic usability concerns. Streamlined cognitive walkthroughs were conducted after documenting heuristics to identify or clarify any deviations from a typical device procedure.

Heuristic Protocol

Heuristics allow evaluators to capture usability issues specific to the intended use environment. Candidate technologies with more than one identified heuristic violation of severity rating 4 were eliminated. All candidate technologies with one or less heuristic violation of severity rating 4 advanced to usability testing with clinicians.

Materials

- Candidate technology (n=1 technology) (simulated clinical scenarios, no patients will be used)
- Three trained, human factors specialists (n = 3 evaluators)
    - Three evaluators are the minimum to identify usability problems; about 63% of problems will be identified with 3 evaluators during a heuristic analysis (Nielsen & Landauer, 1993)
- Evaluators can use any provided documentation available to figure out how to use the candidate technology and complete tasks.

Method

- Follow methods in Nielsen-Schneiderman heuristic analysis (Nielsen, 1992, 1994; Schneiderman, 1998) with additional domain-specific heuristics (Johnston, 2021)
    - Evaluators should consider setup/calibration, clinical use, and maintenance/repair tasks.
    - Evaluators should individually evaluate candidate technology without discussion with other evaluators until all evaluations are complete.
- Record data in table like used in Zhang et al. (2003)
- Assign severity ratings assigned per Nielsen's 1994 paper.
- Aggregate data from all 3 evaluators into a single spreadsheet for data analysis
- Overall pass/fail designations determined by evaluator consensus after reviewing data.
- If the candidate technology receives a pass, usability may be further evaluated using summative usability assessment methods.

List of 14 Nielsen-Schneiderman Heuristics (also in Zhang et al., 2003 for med devices)

1. [Consistency] Consistency and standards. Users should not have to wonder whether different words, situations, or actions mean the same thing. Standards and conventions in product design should be followed.

2. [Visibility] Visibility of system state. Users should be informed about what is going on with the system through appropriate feedback and display of information.

3. [Match] Match between system and world. The image of the system perceived by users should match the model the users have about the system.

4. [Minimalist] Minimalist. Any extraneous information is a distraction and a slow-down.

5. [Memory] Minimize memory load. Users should not be required to memorize a lot of information to carry out tasks. Memory load reduces users' capacity to carry out the main tasks.

6. [Feedback] Informative feedback. Users should be given prompt and informative feedback about their actions.

7. [Flexibility] Flexibility and efficiency. Users always learn and users are always different. Give users the flexibility of creating customization and shortcuts to accelerate their performance.

8. [Message] Good error messages. The messages should be informative enough such that users can understand the nature of errors, learn from errors, and recover from errors.

9. [Error] Prevent errors. It is always better to design interfaces that prevent errors from happening in the first place.

10. [Closure] Clear closure. Every task has a beginning and an end. Users should be clearly notified about the completion of a task.

11. [Undo] Reversible actions. Users should be allowed to recover from errors. Reversible actions also encourage exploratory learning.

12. [Language] Use users' language. The language should be always presented in a form understandable by the intended users.

13. [Control] Users in control. Do not give users impression they are controlled by the systems.

14. [Document] Help and documentation. Always provide help when needed.

Neonatal Equipment for Low-Resource Settings (NELRS) Heuristic Set (Johnston, 2021)

The following are domain-specific heuristics to evaluate neonatal medical devices intended for low-resource settings. They were developed to account for specific usability needs in low-resource settings including cleanability, maintainability and reparability, low workload, minimize discomfort, and access to baby.

15. [Cleanability] Easy to clean. All device surfaces should be easily reachable for thorough cleaning without specialized tools or materials. Neonatal sepsis accounts for a significant percentage of newborn deaths in low-income countries, and medical devices that are difficult to clean could increase infection rates because devices are often used to treat multiple newborns.

16. [Maintainability and reparability] Easy to maintain and repair. The system should be easy to maintain and repair just like any other user interface and task. Device maintenance and repair should require a minimal set of tools and easy-to-follow instructions. If instructions are required, they should be simple, concise, easy to understand, and available at time of maintenance/repair. Users should be able to rely upon a minimal, standard set of tools.

17. [Low workload] Not physical demanding to use. Devices should minimize clinician workload to decrease the risk of fatigue or injury given that hospitals in low-resource settings often treat a high volume of patients relative to the number of clinical staff. Users' physical capabilities and limitations should be considered assuming high patient volumes.

18. [Minimize discomfort] Not cause physical discomfort. Devices should not cause discomfort for either the user or baby, such as repetitive movements, awkward postures, or skin irritation. Systems that cause discomfort may compromise patient care in clinical environments.

19. [Access to baby] Easy to respond to baby. Users should be able to quickly and easily access or remove the baby from the device. Rapid access to newborns during emergency situations is critical.

Severity rating scale for heuristic-identified design deficiencies

If a heuristic is violated, it is given a severity rating based on the following scale (Nielson, 1994):

> 0, not a usability problem at all
> 1, cosmetic problem only; need not be fixed unless extra time is available
> 2, minor usability problem; fixing this should be given low priority
> 3, major usability problem; important to fix; should be given high priority
> 4, usability catastrophe; imperative to fix this before product can be released

As a guideline for rating severity, evaluators should consider the proportion of users who will experience it, the impact it will have on their experience with the product, and whether it will be a problem only the first time a user encounters it, or whether it will persistently bother them. A persistent problem with a major impact that most users will encounter will get the highest severity rating.

Template to document heuristic identified design deficiencies

**Heuristic identified design deficiencies (adapted from Zhang et al (2003)**			
**Candidate Technology**		**Date**	
**Evaluator**		**Pass/Fail**	
**Notes**			
**Places of occurrence**	**Usability problem description**	**Heuristics violated**	**Severity rating**
**Other Observations**			

Cognitive Walkthrough Protocol (Spencer, 2000).

Each evaluator mentally walks through each step of a specified task. For each step, they ask themselves the following questions, noting deficiencies in the interface:

1. Will the user know what to do at this step?
2. If the user does the right thing, will they know they did the right thing?
3. Is the user making progress towards their goal?

This data will be recorded in a table by first listing the step and then including notes about deficiencies in response to the questions above. (Column headings might include step, know what to do, know action was correct, and goal progress.)

**Step #**	**Step Description**	**Will user know what to do at this step?**<br><br>(Yes or no. If no, explain deficiency.)	**If user does the right thing, will they know they did right thing?**<br>(Yes or no. If no, explain deficiency.)	**Is user making progress towards their goal?**<br><br>(Yes or no. If no, explain deficiency.)
**Task**	**Setup device**			
1				
2				
**Task**	**Calibrate device**			
1				
2				

References

Nielsen, J. and Landauer, T.K. (1993) A Mathematical Model of the Finding of Usability Problems, INTERCHI'93.

Nielsen, J. (1992, June). Finding usability problems through heuristic evaluation. In Proceedings of the SIGCHI Conference on Human Factors in Computing Systems(pp. 373–380).

Nielsen, J. (1994). Heuristic evaluation. In J. Nielsen &, R. L. Mack (Eds.), Usability inspection methods. John Wiley & Sons.

Schneiderman, B. (1998). Designing the user interface: Strategies for effective human computer interaction(3rd Ed.). Addison-Wesley.

Johnston J, Asma E, Ziegler Acemyan C, Oden M, Richards-Kortum R, Kortum P. Development and Use of Heuristics to Evaluate Neonatal Medical Devices for Use in Low-Resource Settings. *Journal of User Experience*. 2021;17(1):12-20.

Zhang J, Johnson TR, Patel VL, Paige DL, Kubose T. Using usability heuristics to evaluate patient safety of medical devices. *J Biomed Inform*. 2003;36(1-2):23-30.

Spencer R. The Streamlined Cognitive Walkthrough Method, Working Around Social Constraints Encountered in a Software Development Company. *Proceedings of the SIGCHI on Human Factors in Computing Systems.* 2000:353-359.
