# Supplementary material for: Avoid equipment graveyards: rigorous process to improve identification and procurement of effective, affordable, and usable newborn devices in low-resource hospital settings
Source: BMC Pediatr. 2023 Nov 15;23(Suppl 2):569. doi: 10.1186/s12887-023-04362-x (PMC10652423; doi:10.1186/s12887-023-04362-x)
Supplement: Supplementary file 4 — Additional file 4. Usability methods protocol. Protocol for usability evaluations with human subjects (end users). [file 12887_2023_4362_MOESM4_ESM.pdf]

Usability Evaluation Protocol

All participants enrolled for usability evaluations provided written informed consent as part of protocols reviewed and approved by the appropriate ethics committees.

Defining and Measuring System Usability

The methods in this usability protocol align with;

- IEC 62366-1:2015
- ISO 9241-11
- *Human Factors Design Process for Medical devices* by the American National Standards Institute/Association for the Advancement of Medical Instrumentation (2001)
- Human Factors and Ergonomics Society book on *Usability Assessment: How to measure the usability of products, services, and systems* (Kortum, 2016).

Usability refers to the characteristic of the user interface that facilitates use and establishes;

(1) effectiveness
(2) efficiency
(3) user satisfaction

associated with specific tasks in the intended use environment (section 3.16 of IEC 62366-1:2015

Participants

- A minimum of five participants are needed per product category (e.g., phototherapy, CPAP).
- Participants may include nursing students, nurses, and/or clinicians.

Design

- Each participant will complete tasks with all candidate technologies within a product category.
  - A within-subjects design optimizes statistical power and reduces research time
- Device order will be randomized among participants to account for ordering bias
- Multiple product categories may be evaluated in the same session if it does not become too long (1-2 hours maximum per participant)

Measures

ISO 9241-11 recommends efficiency, effectiveness, and satisfaction to assess system usability.

- Efficiency is the amount of time it takes a person to complete a task, (e.g., setup/calibrate the system for use, take a patient measurement).
  - Time to complete the task is measured in seconds; timing begins when the experimenter directs the participant to begin.
  - Each task will be evaluated independently (e.g. calibration vs. treatment).
- Effectiveness is a user's ability to correctly complete tasks without struggling or making errors.
  - Effectiveness is measured by noting if a participant successfully completed the task (yes or no) and by recording any mistakes made while completing the task.
  - Each task will be evaluated independently.
- Satisfaction captures the extent users were satisfied using the system to complete the task.
  - Satisfaction will be measured with the modified, positively-worded System Usability Scale (SUS).
  - SUS will be completed after all tasks are completed with each candidate technology

Materials

- All candidate technologies for a particular product category that previously (a) met all TPP requirements as tested in laboratory and (b) passed the heuristic and cognitive walkthrough evaluations will undergo usability evaluations with clinical users.
- Systems that are deemed to be unusable or have significant usability problems identified by users in Houston will not move onto testing in Malawi.

Procedures

The procedures are based on the methods presented in Kortum (2016). This book published by the Human Factors and Ergonomics Society presents the gold standard of usability assessment. Its methods are used in industry and academic settings to evaluate numerous types of systems.

- Participants will first complete written IRB-approved informed consent.
- Next an overview of the study will be presented, e.g., "Today you will use several medical technologies to [*insert task, e.g. calibrate a bilirubin device, or measure a baby's bilirubin levels*]. By watching you use these devices and later having you complete some surveys, you will help us learn about which systems are easier and harder to use, in addition to your preferred system."
- Participants will complete basic demographic information including gender, age, ethnicity, nationality, education completed, work experience (type and duration), and comfort with and use of technology.
- Participants will then be shown once how to use each system to complete specified task(s) through a training script or training video.
    - The experimenter will follow a training script or present a video to ensure each participant receives the same training.
    - All training scripts and/or videos will be clinician approved.
    - Participants' questions about how to use the devices will be answered since these are not walk-up and use devices.
- Next, participants will be asked to complete the first task on the device.
    - No patients, blood, bodily fluids or sharps will be used.
    - Mannequins and dolls can be used in place of patients.
- While participants are performing the task, the experimenter will collect the following data:
    - time to complete task,
    - whether the participant completed the task or not,
    - if the participant thinks they successfully completed the task,
    - and any deviations from the procedure shown (including recoverable incidents such as self-corrections).
- This procedure will repeat until all tasks are completed on a particular system.
- Next, the participant will complete the system usability scale (SUS) keeping in mind all the tasks they just performed on the system.
- Afterwards, they will move onto the evaluation of the next candidate technology.
- This process will continue until all systems have been used by the participant, with system orders randomly assigned.
- Last, the participants will complete a final survey, which will include items responded to on a Likert scale to understand overall perceptions and preferences; open ended questions to identify system strengths and weaknesses, along with aspects of the system that need to change or could be improved.

- At the very end of the survey, participants will be asked to choose which system they would want to use while working in the nursery caring for their patients; this will be done for each product category assessed.
- After the final survey is complete, participants will be debriefed (which includes answering their questions and asking them to not talk with their friends about the study), thanked for their time, and compensated for their time.

Outcomes

The summative usability assessment methods described in this document will enable all candidate technologies for a product category to be compared against one another. Using these methods will also generate a list of usability problems per device. These types of data will allow us to understand how user performance and perceptions differ by device.

System Usability Survey

Instructions: For each of the following statements, mark one box that best describes your reactions to the medical device that you just used to complete your task(s).

	Strongly Disagree				Strongly Agree
1. I think I would like to use this medical device frequently.	☐ 1	☐ 2	☐ 3	☐ 4	☐ 5
2. I found the medical device to be simple.	☐ 1	☐ 2	☐ 3	☐ 4	☐ 5
3. I thought the medical device was easy to use.	☐ 1	☐ 2	☐ 3	☐ 4	☐ 5
4. I think that I could use this medical device without the support of a technical person.	☐ 1	☐ 2	☐ 3	☐ 4	☐ 5
5. I found the various functions in this medical device were well integrated.	☐ 1	☐ 2	☐ 3	☐ 4	☐ 5
6. I thought there was a lot of consistency in this medical device.	☐ 1	☐ 2	☐ 3	☐ 4	☐ 5
7. I would imagine that most people would learn to use this medical device very quickly.	☐ 1	☐ 2	☐ 3	☐ 4	☐ 5
8. I found the medical device very intuitive.	☐ 1	☐ 2	☐ 3	☐ 4	☐ 5
9. I felt very confident using the medical device.	☐ 1	☐ 2	☐ 3	☐ 4	☐ 5
10. I was able to start using this medical device without having to learn a lot of things.	☐ 1	☐ 2	☐ 3	☐ 4	☐ 5

Bangor A, Kortum PT, Miller JT. An Empirical Evaluation of the System Usability Scale. *Int J Hum Comput Interact*. 2008;24(6):574-594.
