# Supplementary material for: Avoid equipment graveyards: rigorous process to improve identification and procurement of effective, affordable, and usable newborn devices in low-resource hospital settings
Source: BMC Pediatr. 2023 Nov 15;23(Suppl 2):569. doi: 10.1186/s12887-023-04362-x (PMC10652423; doi:10.1186/s12887-023-04362-x)
Supplement: Supplementary file 5 — Additional file 5. Product Category Report Cards. Report cards by product category with device make and model anonymised. [file 12887_2023_4362_MOESM5_ESM.pdf]

Optimal	Minimal	Does not meet minimal	Unknown

Syringe Pump

Evaluated against Target Product Profile (TPP) as reported by manufacturer

TPP Characteristic	Optimal Requirement	Minimal Requirement	A	B	C	D	E	F	G	H	I	J	K	L	M	N	O	P	Q	R	S	T	U
Intended Use	Treatment of conditions requiring precise administration of drugs and fluids, including but not limited to dextrose solution for hypoglycemia and antibiotics for infection																						
Target Operator	For use in low- and middle-income countries by a wide variety of clinicans, including nurses, clinical officers, and pediatricians																						
Target Population	Neonates (born at any gestational age and require ongoing care)																						
Target Setting	Hospitals in low-resource settings																						
Quality Management	ISO 13485:2016 Medical devices - Quality management systems																						
Regulation	At least one of: CE marking, approved by US FDA etc																						
Benchtop Measurement Accuracy (for Flow Rate)	±1.0%	±3.0%																					
Flow Rate Requirements	0.1-60 mL/hr																						
Occlusion Detection	Continuous adjustment (fully adjustable)	Adjusted based on pre-set (5, 10, 25 psi)																					
Syringe Requirements	Syringe 5-60mL, works with multiple syringe types																						
Drug Library	Yes	No																					
Alarm Characteristics	Visual and auditory																						
Size	Small footprint; portable																						
Weight	< 1.5 kg (without batteries)	<5 kg (without batteries)																					
Instrument Pricing	<$300 ex-works	<$1,000 ex-works																					
Power Source	Mains with rechargable battery																						
Battery	Rechargable battery > 12 hr on single charge	Rechargable battery > 4 hr on single charge																					
Voltage	Model must match the voltage and frequency of the purchasing country's local power grid (e.g., 110-120 VAC at 60 Hz or 220-240 VC at 50 Hz)																						
User Instructions	User manual and additional training materials (checklists, videos, guides) in English and local language. Attached to device with labels and markings where possible	User manual provided in at least one national official language																					
Warranty	5 years	1 year																					
Decontamination	Easy to clean with common disenfecting agents																						
		Purchased for evaluations?	Yes	Yes	Yes	Yes	Yes	Yes	No	No	No	No	No	No	No	No	No	No	No	No	No	No	No

			A	B	C	D	E	F	G–U
Technical	Does device meet all minimal performance characteristics from TPP?	Verified by lab evaluations on =2 devices by 3 unique users				did not test, failed initial heuristics			Did not meet required specifications when evaluated against the TPP
Environmental	Is device rugged, durable, easy to maintain and repair?	Heat							
		Humidity							
		Dust							
		Power							
Usability	Can doctors and nurses use the device with minimal training?	Heuristic evaluation	Pass	Pass	Pass	Fail	Fail	Fail	
		Houston task effectiveness (Infusion)	100%	50%	67%	Failed initial heuristics			
		Houston task effectiveness (Occlusion)	100%	100%	100%				
		Houston SUS <50 50-70 >70	90	71	74				
		Fraction of Houston users indicating preferred device	83%	0%	17%				
		Malawi task effectiveness (Infusion)	17%	0%	17%				
		Malawi task effectiveness (Occlusion)	83%	100%	100%				
		Malawi SUS <50 50-70 >70	76	55	61				
		Fraction of Malawi users indicating preferred device	50%	33%	17%				

			A	B	C	D	E	F	G	H	I	J	K	L	M	N	O	P	Q	R	S	T	U
		NEST Qualified Technology?	Yes	No	No	No	No	No	No	No	No	No	No	No	No	No	No	No	No	No	No	No	No

Optimal	Minimal	Does not meet minimal	Unknown

Bilirubinometer

Evaluated against Target Product Profile (TPP) as reported by manufacturer

TPP Characteristic	Optimal Requirement	Minimal Requirement	A	B	C	D	E	F	G	H	I	J	K	L
Intended Use	Quantification of serum bilirubin in neonates for the diagnosis and management of jaundice at the patient's bedside													
Target Operator	For use in low and middle income countries by a wide variety of clinicans, including nurses, clinical officers, and pediatricians													
Target Population	Neonates (born at any gestational age and require ongoing care)													
Target Setting	Hospitals in low-resource settings													
Quality Management	ISO 13485:2016 medical devices - Quality management systems -- Requirements for regulatory purposes													
Regulation	At least one of: CE marking, approved by US FDA or another stringent regulatory body of a founding member of IMDRF (e.g., Japan or Australia or Canada)													
Linear Range	0-40 mg/dL (0-684 umol/L)	5-30 mg/dL (85.5 - 513 umol/L)												
Accuracy	+/- 10% from 5-30mg/dL	+/- 20% from 5-30mg/dL												
Results Format	Quantitative across whole linear range													
Results Units	Must display mg/dL and umol/L (shall have ability to select or switch between either)													
Precision	4% CV	15% CV												
Sample	Whole blood heel-stick sample <50uL; does not require user to separate serum/plasma using a centrifuge													
Calibration	No Calibration	Minimal user calibration required												
Kit Stability & Storage	Stable for >12 months with harsh ambient conditions (temperature 5-45 °C, humidity 15% to 95%, dusty air, elevation >=2000 meters) and transport stress (48h with fluctuations up to 50°C and down to 0°C)	Stable for 12 months with harsh ambient conditions (temperature 10-40 °C, humidity 15%-95% elevation up to 2000 meters) and transport stress (48h with fluctuations up to 50°C and down to 0°C)												
Equipment Required	Small, portable or hand-held device; device-free/disposable preferred; does not require centrifuge	Small, table-top device; portable device optional; does not require centrifuge												
Instrument Pricing	< $200 ex-works	<$800 ex-works												
Consumable Pricing	<$0.50 per test ex-works	$1.50 per test ex-works												
Power Source	No power required	Mains with rechargable battery												
Battery	None (i.e. a disposable test that requires no electricity)	Rechargable battery >100 tests on a single charge.												
Voltage	None	Model must match the voltage and frequency of the purchasing country's local power grid (e.g., 110-120 VAC at 60 Hz or 220-240 VAC at 50 Hz)												
		Purchased for evaluations?	Yes	Yes	Yes	No	No	No	No	No	No	No	No	No
Technical	Does device meet all minimal performance characteristics from TPP?	Verified by lab evaluations on n = 2 devices by 3 unique users				Did not meet required specifications when evaluated against the TPP								
Environmental	Is device rugged, durable, easy to maintain and repair?	Heat												
		Humidity												
		Dust	Did not test											
		Power												
Usability	Can doctors and nurses use the device with minimal training?	Heuristic evaluation												
		Houston task effectiveness (Measure)	83%	33%	66%									
		Houston task effectiveness (Calibrate)	N/A	83%	83%									
		Houston SUS <50 50-70 >70	87	60	63									
		Fraction of Houston users indicating preferred device	100%	0%	0%									
		Malawi task effectiveness (Measure)	80%	60%	20%									
		Malawi task effectiveness (Calibrate)	N/A	40%	40%									
		Malawi SUS <50 50-70 >70	95	76	48									
		Fraction of Malawi users indicating preferred device	80%	20%	0%									
		NEST Qualified Technology?	Yes	Yes	No	No	No	No	No	No	No	No	No	No

Optimal	Minimal	Does not meet minimal	Unknown

Phototherapy

Evaluated against Target Product Profile (TPP) as reported by manufacturer

TPP Characteristic	Optimal Requirement	Minimal Requirement	A	B	C	D	E	F	G	H	I	J	K	L	M	N	O	P	Q
Intended Use	Treatment of hyperbilirubinemia in neonates																		
Target Operator	For use in low- and middle-income countries by a wide variety of clinicans, including nurses, clinical officers, and pediatricians																		
Target Population	Neonates (born at any gestational age and require ongoing care)																		
Target Setting	Hospitals in low-resource settings																		
Quality Management	ISO 13485:2016 Medical devices - Quality management systems																		
Regulation	At least one of: CE marking, approved by US FDA or another strigent regulatory body of a founding member of IMDRF (e.g., Japan or Australia or Canada)																		
Irradiance	Standard Phototherapy: 8-10 AND Intensive Phototherapy: >30 uW/cm2/nm																		
Effective treatment area	>2000 cm2	>1300 cm2																	
Peak wavelength	430-490 nm																		
Light Source	LED																		
Bulb Lifetime	60,000 hours	44,000 hours																	
Ease of Replacing Bulbs	Capable of being replaced by a technician with minimal training and basic tools (screwdrivers)																		
Irradiance Meter	Included	Available																	
Instrument Pricing	< $400 ex-works	< $1000 ex-works																	
Power Source	Mains with battery backup	Mains power																	
Battery	Provides battery backup	Mains Power																	
Voltage	Model must match the voltage and frequency of the purchasing country's local power grid (e.g., 110-120 VAC at 60 Hz or 220-240 VAC at 50 Hz)																		
User instructions	User manual and additional training materials (checklists, videos, guides) in at least one national language for the country of intended use. Attached to device with labels and markings where possible.	User manual provided in at least one national official language																	
Warranty	5 years	1 year																	
		Purchased for evaluations?	Yes	Yes	Yes	No	No	No	No	No	No	No	No	No	No	No	No	No	No
Technical	Does device meet all minimal performance characteristics from TPP?	Verified by lab evaluations on n=2 devices by 3 users																	
Environmental	Is device rugged, durable, easy to maintain and repair?	Heat																	
		Humidity																	
		Dust																	
		Power																	
Usability	Can doctors and nurses use the device with minimal training?	Heuristic evaluation	Pass	Pass	Pass														
		Houston task effectiveness (setup & use in high-intensity mode)	100%	100%	100%	Did not meet required specifications when evaluated against the TPP													
		Houston SUS <50 50-70 >70	87	93	97														
		Fraction of Houston users indicating preferred device	25%	25%	50%														
		Malawi task effectiveness (setup & use in high-intensity mode)	83%	75%	not tested														
		Malawi SUS <50 50-70 >70	79	83	not tested														
		Fraction of Malawi users indicating preferred device	42%	58%	0%														
		Qualified Technology?	Yes	Yes	Yes	No	No	No	No	No	No	No	No	No	No	No	No	No	No

Optimal	Minimal	Does not meet minimal	Unknown

Glucometer

Evaluated against Target Product Profile (TPP) as reported by manufacturer

TPP Characteristic	Optimal Requirement	Minimal Requirement	A	B	C	D	E	F	G	H	I	J	K	L	M
Intended Use	Quantitative measurment of blood glucose for diagnosis and management of neonates														
Target Operator	For use in low- and middle-income countries by a wide variety of clinicans, including nurses, clinical officers, and pediatricians														
Target Population	Neonates (born at any gestational age and require ongoing care)														
Target Setting	Hospitals in low-resource settings, but, may be used in health facilites based on country guidelines	Hospitals in low-resource settings													
Quality Management	ISO 13485:2016 Medical devices - Quality management systems - Requirements for regulatory purposes														
Regulation	At least one of: CE marking, approved by US FDA or another strigent regulatory body of a founding member of IMDRF (e.g., Japan or Australia or Canada)														
Linear range	0-50 mmol/L (0 - 900 mg/dL)	0-20 mmol/L (0 - 360 mg/dL)													
Accuracy	±6% across whole range ± 0.2 mmol/L at 2.5 mmol/L	±8% across whole range ± 0.2 mmol/L at 3 mmol/L													
Results Format	Quantitative across whole linear range (should be able to switch between mg and mmol)														
Results Units	mg/dL OR mmol/L														
Precision	+-2% or 2.5 mg/dL, whichever is greater														
Sample	Whole blood heel stick sample <5 uL	Whole blood heel stick <50 uL													
Calibration	No calibration	Minimal user calibration required													
Kit Stability & Storage	Stable for >12 months with harsh ambient conditions (temperature 5-45 °C, humidity 15% to 95%, dusty air, elevation >=2000 meters) and transport stress (48h with fluctuations up to 50°C and down to 0°C)	Stable for 12 months with harsh ambient conditions (temperature 10-40 °C, humidity 15%-95% elevation up to 2000 meters) and transport stress (48h with fluctuations up to 50°C and down to 0°C)													
Equipment Required	Small, portable or hand-held device; device-free/disposable preferred	Small, table-top device; portable device optional													
Instrument Pricing	<$30 ex-works														
Consumable Pricing	$0.05 per test ex-works, ideally with generic strips	$0.20 per test ex-works													
Power Source	No power required	Mains with rechargeable battery													
Battery	None (i.e. a disposable test that requires no electricity)	Rechargeable battery, > 100 tests on a single charge													
Voltage	None.	Model must match the voltage and frequency of the purchasing													
		Purchased for evaluations?	Yes	Yes	Yes	No	No	No	No	No	No	No	No	No	No
Technical	Does device meet all minimal performance characteristics from TPP?	Verified by lab evaluations on n = 2 devices by 3 unique users				Did not meet required specifications when evaluated against the TPP									
Environmental	Is device rugged, durable, easy to maintain and repair?	Heat													
		Humidity													
		Dust	Did not test												
		Power		N/A											
Usability	Can doctors and nurses use the device with minimal training?	Heuristic Evaluation													
		Houston task effectiveness (Measure)	83%	100%	100%										
		Houston SUS <50 50-70 >70	83	93	79										
		Fraction of Houston users indicating preferred device	33%	67%	0%										
		Malawi task effectiveness (Measure)	Not included in evals	64%	57%										
		Malawi SUS <50 50-70 >70		79	67										
		Fraction of Malawi users indicating preferred device		36%	64%										
		NEST Qualified Technology?	Yes	Yes	No	No	No	No	No	No	No	No	No	No	No

Optimal | Minimal | Does not meet minimal | Unknown

Hemoglobinometer

Evaluated against Target Product Profile (TPP) as reported by manufacturer

TPP Characteristic	Optimal Requirement	Minimal Requirement	A	B	C	D	E	F	G	H	I	J	K	L	M	N	O	P	Q	R	S	T
Intended Use	Quantitative determiniation of hemoglobin in capillary, venous, or arterial whole blood																					
Target Operator	For use in low- and middle-income countries by a wide variety of clinicans, including nurses, clinical officers, and pediatricians																					
Target Population	Neonates (born at any gestational age and require ongoing care)																					
Target Setting	Hospitals in low-resource settings																					
Quality Management	ISO 13485:2016 Medical devices - Quality management systems																					
Regulation	At least one of: CE marking, approved by US FDA or another strigent regulatory body of a founding member of IMDRF (e.g., Japan or Australia or Canada)																					
Linear Range	0-25 g/dL	4.5-25 g/dL																				
Accuracy	+-1 g/dL	+-1.75 g/dL																				
Results Format	Quantitative across whole linear range	Quantitative; semi-quantitative below 5 or above 25 g/dL																				
Result Units	g/dL OR g/L																					
Precision	1.5% CV	2% CV																				
Sample	Whole blood heel-stick sample <10 µL	Whole blood heel-stick sample <25 µL																				
Number of Steps	No more than 1-3 steps (requiring operator intervention)	No more than 4-6 steps (requiring operator intervention)																				
Calibration	No calibration	Minimal user calibration required																				
Kit Stability & Storage	Stable for >12 months with harsh ambient conditions (temperature 5-45 °C, humidity 15% to 95%, dusty air, elevation >=2000 meters) and transport stress (48h with fluctuations up to 50°C and down to 0°C)	Stable for 12 months with harsh ambient conditions (temperature 10-40 °C, humidity 15%-95% elevation up to 2000 meters) and transport stress (48h with fluctuations up to 50°C and down to 0°C)																				
Equipment Required	Small, portable or hand-held device; device-free/disposable preferred	Small, table-top device; portable device optional																				
Instrument Pricing	< $200 ex-works	< $300 ex-works																				
Consumable Pricing	$0.05 per test ex-works	$0.50 per test ex-works																				
Power Source	No power required	Mains with rechargable battery																				
Voltage	None	Model must match the voltage and frequency of the purchasing country's local power grid (e.g., 110-120 VAC at 60 Hz or 220-240 VAC at 50 Hz)																				
		Purchased for evaluations?	Yes	Yes	Yes	Yes	Yes	Yes	Yes	No	No	No	No	No	No	No	No	No	No	No	No	No
Technical	Does device meet all minimal performance characteristics from TPP?	Verified by lab evaluations on n = 2 devices by 3 unique users								Did not meet required specifications when evaluated against the TPP												
Environmental	Is device rugged, durable, easy to maintain and repair?	Heat						did not test, failed technical testing														
		Humidity																				
		Dust	Did not test																			
		Power				N/A	N/A															
Usability	Can doctors and nurses use the device with minimal training?	Heuristic Evaluation																				
		Houston task effectiveness (Measure)	33%	Did not test	100%	100%	Did not test															
		Houston SUS <50 50-70 >70	62		92	62																
		Fraction of Houston users indicating preferred device	17%		83%	0%																
		Malawi task effectiveness (Measure)	100%		67%	50%																
		Malawi SUS <50 50-70 >70	87		65	62																
		Fraction of Malawi users indicating preferred device	100%		0%	0%																
		NEST Qualified Technology?	Yes	Yes	No	No	No	No	No	No	No	No	No	No	No	No	No	No	No	No	No	No

Optimal	Minimal	Does not meet minimal	Unknown

CPAP

Evaluated against Target Product Profile (TPP) as reported by manufacturer

TPP Characteristic	Optimal Requirement	Minimal Requirement	A	B	C	D	E	F	G	H	I	J	K	L	M	N	O	P	Q	R
Intended Use	To treat respiratory distress and other forms of respiratory illness in infants up to one year of age																			
Target Operator	For use in low- and middle-income countries by a wide variety of clinicans, including nurses, clinical officers, and pediatricians																			
Target Population	Neonates (born at any gestational age and require ongoing care)																			
Target Setting	Hospitals in low-resource settings																			
Quality Management	ISO 13485:2016 Medical devices - Quality management systems																			
Regulation	At least one of: CE marking, approved by US FDA or another strigent regulatory body of a founding member of IMDRF (e.g., Japan or Australia or Canada)																			
Flow driver	Integrated (on-board air compressor)																			
Oxygen Flow Capacity	0-10 L/min																			
Pressure	5-8 cm H2O																			
Total (blended) Flow	0-10 L/min																			
Humidification	Yes, Heated Humidification	None																		
Alarms	Audio/Visual Power, low-flow, low-pressure	Audio Power																		
Accessories	Non-proprietary	Proprietary																		
Consumables	Reusable	Available																		
Instrument Pricing	<$1,000 ex-works	<$2,000 ex-works																		
Consumable Pricing	<$10 / patient ex-works	<$15 per patient ex-works																		
Power Source	Mains with battery backup	Mains Power																		
Battery	Rechargeable intergrated battery, >6 hours on a single charge	None																		
Voltage	Model must match the voltage and frequency of the purchasing country's local power grid (e.g., 110-120 VAC at 60 Hz or 220-240 VAC at 50 Hz)																			
User Instructions	User manual and additional training materials (checklists, videos, guides) in at least one national language for the country of intended use. Attached to device with labels and markings where possible.	User manual provided in at least one national official language																		
Warranty	5 years	1 year																		
		Purchased for evaluations?	Yes	Yes	Yes	No	No	No	No	No	No	No	No	No	No	No	No	No	No	No
Technical	Does device meet all minimal performance characteristics from TPP?	Verified by lab evaluations on n = 2 devices by 3 unique users																		
Environmental	Is device rugged, durable, easy to maintain and repair?	Heat																		
		Humidity																		
		Dust																		
		Power																		
Usability	Can doctors and nurses use the device with minimal training?	Heuristic Evaluation																		
		Houston task effectiveness (Setup)	83%	100%	not included in evals															
		Houston task effectiveness (Titration)	100%	66%																
		Houston task effectiveness (Hat/prong)	50%	100%		Did not meet required specifications when evaluated against the TPP														
		Houston SUS <50 50-70 >70	48	58																
		Fraction of Houston users indicating preferred device	50%	50%																
		Malawi task effectiveness (Setup)	50%	50%	0%															
		Malawi task effectiveness (Titration)	0%	25%	0%															
		Malawi task effectiveness (Hat/prong)	25%	75%	75%															
		Fraction of Malawi users indicating preferred device	25%	75%	0%															
		Malawi SUS <50 50-70 >70	71	74	29															
		NEST Qualified Technology?	Yes	Yes	No	No	No	No	No	No	No	No	No	No	No	No	No	No	No	No

Optimal	Minimal	Does not meet minimal	Unknown

Flow Splitter

Evaluated against Target Product Profile (TPP) as reported by manufacturer

TPP Characteristic	Optimal Requirement	Minimal Requirement	A	B	C	D
Intended Use	To allow multiple patients to receive individually adjusted flow rates from a single source of oxygen					
Target Operator	For use in low- and middle-income countries by a wide variety of clinicans, including nurses, clinical officers, and pediatricians					
Target Population	Neonates (born at any gestational age and require ongoing care)					
Target Setting	Hospitals in low-resource settings, but, may be used in health facilities based on country guidelines	Hospitals in low-resource settings				
Quality Management	ISO 13485:2016 Medical devices - Quality management systems - Requirements for regulatory purposes					
Regulation	At least one of: CE marking, approved by US FDA or another strigent regulatory body of a founding member of IMDRF (e.g., Japan or Australia or Canada)					
Air Flow per Patient	0-2 L/min					
Flow Control	Each patient has individually controlled flow rate					
Number of Outputs	5	2				
Indication	Each flow rate has a visual indicator					
Instrument Pricing	<$100 ex-works	<$600 ex-works				
Maintenance	No/minimal maintenance					
		Purchased for evaluations?	Yes	Yes	Yes	No
Technical	Does device meet all minimal performance characteristics from TPP?	Verified by lab evaluations on n = 2 devices by 3 unique users				Did not meet required specifications when evaluated against the TPP
Environmental	Is device rugged, durable, easy to maintain and repair?	Heat				
		Humidity				
		Dust				
		Power	N/A	N/A	N/A	
Usability	Can doctors and nurses use the device with minimal training?	Heuristic Evaluation				
		Houston task effectiveness	80%	100%	Did not test	
		Houston SUS <50 50-70 >70	58	76		
		Fraction of Houston users indicating preferred device	0%	100%		
		Malawi task effectiveness	Did not test			
		Malawi SUS <50 50-70 >70				
		Fraction of Malawi users indicating preferred device				
		NEST Qualified Technology?	Yes	Yes	Yes	No

Optimal	Minimal	Does not meet minimal	Unknown

Oxygen Concentrator

Evaluated against Target Product Profile (TPP) as reported by manufacturer

TPP Characteristic	Optimal Requirement	Minimal Requirement	A	B	C	D	E	F	G	H	I	J	K	L	M	N	O	P	Q	R	S	t	U	V	W	X
Intended Use	To provide medical oxygen for use in a healthcare setting																									
Target Operator	For use in low- and middle-income countries by a wide variety of clinicans, including nurses, clinical officers, and pediatricians																									
Target Population	Neonates (born at any gestational age and require ongoing care)																									
Target Setting	Hospitals in low-resource settings, but, may be used in health facilites based on country guidelines	Hospitals in low-resource settings																								
Quality Management	ISO 13485:2016 Medical devices - Quality management systems																									
Regulation	At least one of: CE marking, approved by US FDA or another strigent regulatory body of a founding member of IMDRF (e.g., Japan or Australia or Canada)																									
Flow Meter	At least 2 with each 0 to 10 LPM flow meter, min incremental 0.5 LPM	At least 1 [flow meter] with 0 to 10 LPM flow meter, min incremental 0.5 LPM																								
Minimal Flow Rate	0.5 LPM (if used without a flow splitter)	2 LPM																								
Flow Rate	10 LPM	8-10 LPM																								
Time to reach 95% of specified performance	<5 Min																									
Oxygen Purity	93%+-3%																									
Alarms	Visual and auditory alarms																									
Indicators	Clearly labeled or marked with pictures and language. Audible alerts and diagnostic indicator where possible.	UI is easy to understand, numbers and displays are clearly visible.																								
Mobility	Whole unit moveable with wheels on at least two feet																									
Oxygen Monitor	Visual and audible status, preferably with color coding for early warning	Visual and audible status																								
Oxygen Outlet	Recessed, replaceable metal barbs.																									
Noise Level	≤ 50 decibels; low as posisble																									
Weight	<30kg																									
Durability and Robustness	Harsh ambient condition, temperature 5-45 °C, humidity 15% to 95%, dusty air, elevation ≥ 2000 meters	Temperature 10-40 °C, humidity 15%-95% elevation up to 2000 meters																								
Usage Meter	Non-resettable digital or analog meter displaying cumulative hours of operation																									
Instrument Pricing	<$500 ex-works	<$1600 ex-works																								
Power Source	Mains Power	Mains Power																								
Power Consumption	< 275W at 5 PLM	Scales with delivery output — i.e., consumes less power at lower flow rates.																								
Voltage	Model must match the voltage and frequency of the purchasing country's local power grid (e.g., 110-120 VAC at 60 Hz or 220-240 VAC at 50 Hz)																									
User Instructions	User manual and additional training materials (checklists, videos, guides) in at least one national offical language for the country of intended use. Attached to device with labels and markings where possible.	Instruction manual provided in at least one national offical language																								
User Skill Levell	Minimal to none																									
Warranty	5 years	1 year																								
Decontamination	Reduce recessed areas and need for specialized cleaning procedures or products	Easy to clean flat surfaces, compatible with common disinfecting agents																								
Preventive Maintenance Interval	Should not need preventative maintenance more than once a year	Should not need preventative maintenance more than 4 times a year (quarterly)																								
Technical Skill Maintenance	Minimally trained technician	Trained technician with training in basic operation and maintenance																								
Cleaning Interval	None required.	Weekly filter cleaning.																								
Tools Required	No specialized tools required	Minimal specialized tools required for sieve bed and filter replacement																								
Filters	Replaceable washable reusable																									
		Purchased for evaluations?	Yes	Yes	Yes	No	No	No	No	No	No	No	No	No	No	No	No	No	No	No	No	No	No	No	No	No

			A	B	C	D–X
Technical	Does device meet all minimal performance characteristics from TPP?	Verified by lab evaluations on n = 2 devices by 3 unique users				Did not meet required specifications when evaluated against the TPP
Environmental	Is device rugged, durable, easy to maintain and repair?	Heat				
		Humidity				
		Dust				
		Power				
Usability	Can doctors and nurses use the device with minimal training?	Heuristic Evaluation				
		Houston task effectiveness (Setup/report)	83%	83%	67%	
		Houston task effectiveness (Alarm)	100%	100%	100%	
		Houston SUS <50 50-70 >70	83	87	71	
		Fraction of Houston users indicating preferred device	33%	0%	67%	
		Malawi task effectiveness (Setup/report)	Evaluations were not completed due to COVID-19 pandemic			
		Malawi task effectiveness (Alarm)				
		Malawi SUS <50 50-70 >70				
		Fraction of Malawi users indicating preferred device				

			A	B	C	D	E	F	G	H	I	J	K	L	M	N	O	P	Q	R	S	t	U	V	W	X
		NEST Qualified Technology?	Yes	Yes	Yes	No	No	No	No	No	No	No	No	No	No	No	No	No	No	No	No	No	No	No	No	No

Optimal	Minimal	Does not meet minimal	Unknown

Pulse Oximeter

Evaluated against Target Product Profile (TPP) as reported by manufacturer

TPP Characteristic	Optimal Requirement	Minimal Requirement	A	B	C	D	E	F	G	H	I	J	K	L	M	N	O	P	Q	R	S	T	U	V	W	X	Y
Intended Use	To continuously monitor oxygen saturation (SpO2) and pulse rate (PR) for neonatal patients																										
Target Operator	For use in low- and middle-income countries by a wide variety of clinicans, including nurses, clinical officers, and pediatricians																										
Target Population	Neonates (born at any gestational age and require ongoing care)																										
Target Setting	Hospitals in low-resource settings, but, may be used in health facilites based on country	Hospitals in low-resource settings																									
Quality Management	ISO 13485:2016 Medical devices - Quality management systems -- Requirements for regulatory purposes																										
Regulation	At least one of: CE marking, approved by US FDA or another stringent regulatory body of a founding member of IMDRF (e.g., Japan or Australia or Canada)																										
Pulse rate	25-250 bpm	30-240 bpm																									
Pulse rate accuracy	+/-3 bpm																										
Pulse rate resolution	1 bpm																										
SPO2 accuracy	+-2%	+-3%																									
SPO2 range	0-100%	70-100%																									
Alarm characteristics	Visual and auditory	Auditory																									
Alarm limits - PR	Adjustable	80-180 bpm OR 100-180 bpm																									
Alarm limits - SpO2	Adjustable																										
Continuous measurement	Yes																										
Patient interface	Neonate specific, biocompatible and reusable.																										
Size	Easily moveable, not pocketable, can be secured	Handheld with dock																									
Weight	<500 g, portable																										
Consumables	>12 months before required	>6 months before required with 2 neonatal probes included in package																									
Instument Pricing	< $150 ex-works	< $250 ex-works																									
Consumable Pricing	< $50/year ex-works (two probes)	< $80 per year ex-works (two probes)																									
Power Source	Mains with rechargeable battery																										
Battery	Rechargeable battery, >24hr on single charge	Rechargeable battery, >6hr on single charge																									
Voltage	None	Model must match the voltage and frequency of the purchasing country's local power grid (e.g., 110-120 VAC at 60 Hz or 220-240 VAC at 50 Hz)																									
User instructions	User manual and additional training materials (checklists, videos, guides) in at least one national official language for the country intended use. Attached to device with labels and markings where possible	User manual provided in at least one national official language																									
Training Required	Minimal																										
Warranty	5 years	1 year																									
Decontamination	Easy to clean with common disenfection agents																										
Usage Meter	Digitally stored record displaying cumulative hours of operation	Digitally stored record displaying 50 previous readings or >50 hours																									
		Purchased for evaluations?	Yes	Yes	Yes	Yes	Yes	No	No	No	No	No	No	No	No	No	No	No	No	No	No	No	No	No	No	No	No
Technical	Does device meet all minimal performance characteristics from TPP?	Verified by lab evaluations on n = 2 devices by 3 unique users				Did not test, failed initial heuristics																					
Environmental	Is device rugged, durable, easy to maintain and repair?	Heat																									
		Humidity																									
		Dust	Did not test																								
		Power																									
Usability	Can doctors and nurses use the device with minimal training?	Heuristic Evaluation																									
		Houston task effectiveness (Setup/report)	83%	100%	83%																						
		Houston task effectiveness (Alarm)	100%	100%	83%																						
		Houston SUS <50 50-70 >70	82	90	45			Did not meet required specifications when evaluated against the TPP																			
		Fraction of Houston users indicating preferred device	33%	67%	0%	Failed initial heuristics with LMIC clinicans																					
		Malawi task effectiveness (Setup/report)	83%	83%	58%																						
		Malawi task effectiveness (Alarm)	42%	42%	8%																						
		Malawi SUS <50 50-70 >70	80	80	52																						
		Fraction of Malawi users indicating preferred device	42%	42%	16%																						
		NEST Qualified Technology?	Yes	Yes	No	No	No	No	No	No	No	No	No	No	No	No	No	No	No	No	No	No	No	No	No	No	No

Optimal	Minimal	Does not meet minimal	Unknown

Suction Pump

Evaluated against Target Product Profile (TPP) as reported by manufacturer

TPP Characteristic	Optimal Requirement	Minimal Requirement	A	B	C	D	E	F	G
Intended Use	Aspirations and removal of secretions, bodily fluids and foreign objects from a patient's airway or respiratory support system in the nasal, pharyngeal and								
Target Operator	For use in low- and middle-income countries by a wide variety of clinicans, including nurses, clinical officers, and pediatricians								
Target Population	Neonates (born at any gestational age and require ongoing care)								
Target Setting	Hospitals in low-resource settings								
Quality Management	ISO 13485:2016 Medical devices - Quality management systems								
Regulation	At least one of: CE marking, approved by US FDA or another atrigent regulatory body of a founding member of IMDRF (e.g., Japan or Australia or Canada)								
Pressure	60-120 mm Hg with continuous adjustment								
Bottle Capacity	1L								
Noise Level	As low as possible								
Cleaning	Collection vessel easy to clean reusable								
Maintenance	No maintenance or lubrication								
Operation Mode	Adjustable to neonatal setting (60-100 mm Hg)								
Instrument Pricing	<$100 ex-works	<$250 ex-works							
Power Source	Mains Power								
Voltage	Model must match the voltage and frequency of the purchasing country's local power grid (e.g., 110-120 VAC at 60 Hz or 220-240 VAC at 50 Hz)								
User instructions	User manual and additional training materials (checklists, videos, guides) in at least one national official language for the country of intended use. Attached to device with labels and markings where possible.	User manual provided in at least one national official language							
Warranty	5 years	1 year							
		Purchased for evaluations?	Yes	Yes	Yes	No	No	No	No
Technical	Does device meet all minimal performance characteristics from TPP?	Verified by lab evaluations on n = 2 devices by 3 unique users				Did not meet required specifications when evaluated against the TPP			
Environmental	Is device rugged, durable, easy to maintain and repair?	Heat							
		Humidity							
		Dust							
		Power							
Usability	Can doctors and nurses use the device with minimal training?	Heuristic Evaluation							
		Houston task effectiveness (Setup)	100%	33%	100%				
		Houston task effectiveness (Use)	100%	100%	100%				
		Houston SUS <50 50-70 >70	80	68	84				
		Fraction of Houston users indicating preferred device	0%	0%	100%				
		Malawi task effectiveness (Setup)	75%	50%	Did not test				
		Malawi task effectiveness (Use)	100%	100%					
		Malawi SUS <50 50-70 >70	85	85					
		Fraction of Malawi users indicating preferred device	50%	50%					
		NEST Qualified Technology?	Yes	No	No	No	No	No	No

Optimal | Minimal | Does not meet minimal | Unknown

Radiant Warmer

Evaluated against Target Product Profile (TPP) as reported by manufacturer

TPP Characteristic	Optimal Requirement	Minimal Requirement	A	B	C	D	E	F	G	H	I	J	K	L	M	N
Intended Use	Treatment and prevention of hypothermia in neonates requiring intensive thermal care															
Target Operator	For use in low- and middle-income countries by a wide variety of clinicans, including nurses, clinical officers, and pediatricians															
Target Population	Neonates (born at any gestational age and require ongoing care)															
Target Setting	Hospitals in low-resource settings															
Quality Management	ISO 13485:2016 Medical devices - Quality management systems															
Regulation	At least one of: CE marking, approved by US FDA or another strigent regulatory body of a founding member of IMDRF (e.g., Japan or Australia or Canada)															
Benchtop Measurement Accuracy	±0.1°C															
Clinical Measurement Accuracy	±0.3°C															
Stability	< 0.5°C															
Includes Timer	Yes															
Includes Scale	Yes	No														
Mobility	Has wheels; can be moved by one person															
Time to Indicate Accurate Temperature	< 1 minute	< 90 seconds														
Uniformity	< 1 °C															
Alarm Characteristics	Visual and auditory															
Alarm Limits	Adjustable	36.5-37.5°C														
Operating Temperature	Harsh ambient condition, temperature 5-45 °C, humidity 15% to 95%, dusty air, elevation >=2000 meters	Harsh ambient condition, temperature 10-40 °C, humidity 15% to 95%, dusty air, elevation up to 2000 meters														
Patient Interface	Interface is biocompatible and resusable	Interface is biocompatible														
Patient Accessibility and Visibility	Patient is visible and accessible to healthcare worker															
Temperature Control	Based upon infant's temperature and includes fail-safe mode															
Consumables	> 12 months before required	> 6 months before required														
Instrument Pricing	< $500 ex-works	< $1,500 ex-works														
Consumable Pricing	< $50 per year ex-works (includes two probes)	< $100 per year ex-works (includes two probes)														
Power Source	Mains Power	Mains Power														
Power Consumption	< 250W maximum	< 800W maximum														
Voltage	Model must match the voltage and frequency of the purchasing country's local power grid (e.g., 110-120 VAC at 60 Hz or 220-240 VAC at 50 Hz)															
User Instructions	User manual and additional training materials (checklists, videos, guides) in at least one national official language for the country of intended use. Attached to device with labels and markings where possible.	User manual provided in at least one national official language														
Warranty	5 years	1 year														
Decontamination	Easy to clean with common disinfecting agents															
		Purchased for evaluations?	Yes	Yes	No	No	No	No	No	No	No	No	No	No	No	No
Technical	Does device meet all minimal performance characteristics from TPP?	Verified by lab evaluations on n = 2 devices by 3 unique users			Did not meet required specifications when evaluated against the TPP											
Environmental	Is device rugged, durable, easy to maintain and repair?	Heat														
		Humidity														
		Dust														
		Power														
Usability	Can doctors and nurses use the device with minimal training?	Heuristic Evaluation														
		Houston task effectiveness (Prewarm)	0%	67%												
		Houston task effectiveness (Automatic)	100%	100%												
		Houston task effectiveness (Read/report)	100%	100%												
		Houston task effectiveness (Alarm)	83%	83%												
		Houston SUS <50 50-70 >70	78	80												
		Fraction of Houston users indicating preferred device	33%	67%												
		Malawi task effectiveness (Prewarm)	0%	17%												
		Malawi task effectiveness (Automatic)	33%	67%												
		Malawi task effectiveness (Read/report)	83%	100%												
		Malawi task effectiveness (Alarm)	83%	17%												
		Malawi SUS <50 50-70 >70	63	63												
		Fraction of Malawi users indicating preferred device	50%	50%												
		NEST Qualified Technology?	Yes	Yes	No	No	No	No	No	No	No	No	No	No	No	No

Optimal | Minimal | Does not meet minimal | Unknown

Temperature Monitor

Evaluated against Target Product Profile (TPP) as reported by manufacturer

TPP Characteristic	Optimal Requirement	Minimal Requirement	A	B	C	D	E	F	G	H	I	J	K	L	M	N	O	P	Q	R	S	T	U	V	W	X	Y	Z	AA	BB	CC	DD
Intended Use	To provide ongoing diagnosis and monitoring of treatment of hypo- and hyperthermia																															
Target Operator	For use in low- and middle-income countries by a wide variety of clinicans, including nurses, clinical officers, and pediatricians																															
Target Population	Neonates (born at any gestational age and require ongoing care)																															
Target Setting	Hospitals in low-resource settings																															
Quality Management	ISO 13485:2016 Medical devices - Quality management systems																															
Regulation	At least one of: CE marking, approved by US FDA or another atrigent regulatory body of a founding member of IMDRF (e.g., Japan or Australia or Canada)																															
Benchtop Measurement Accuracy	±0.1°C																															
Clinical Measurement Accuracy	±0.2 ^{o}C	±0.3 C																														
Time to Indicate Accurate Temperature	< 60 seconds	< 90 seconds																														
Alarm Characteristics	Visual and Auditory																															
Alarm Limits	Adjustable	36.5°C-37.5°C																														
Patient Interface	Interface is biocompatible and reusable	Interface is biocompatible																														
Size	Small footprint; can be left at bedside																															
Weight	<500 grams																															
Consumables	> 12 months before required	> 6 months before required																														
Instrument Pricing	<$100 ex-works	<$200 ex-works																														
Consumable Pricing	<$50 per year ex-works																															
Power Source	Mains with rechargeable battery																															
Battery	Rechargeable battery, >24hrs on a single charge	Rechargeable battery, >6hrs on a single charge																														
Voltage	Model must match the voltage and frequency of the purchasing country's local power grid (e.g., 110-120 VAC at 60 Hz or 220-240 VAC at 50 Hz)																															
User instructions	User manual and additional training materials (checklists, videos, guides) in at least one national official language for the country of intended use. Attached to device with labels and markings where possible.	User manual provided in at least one national official language																														
Warranty	5 years	1 year																														
Decontamination	Easy to clean with common disinfecting agents																															
Purchased for evaluations?			No	No	No	No	No	No	No	No	No	No	No	No	No	No	No	No	No	No	No	No	No	No	No	No	No	No	No	No	No	No
Technical	Does device meet all minimal performance characteristics from TPP?	Verified by lab evaluations on n = 2 devices by 3 unique users	Did not meet required specifications when evaluated against the TPP																													
Environmental	Is device rugged, durable, easy to maintain and repair?	Heat																														
		Humidity																														
		Dust																														
		Power																														
Usability	Can doctors and nurses use the device with minimal training?	Houston Satisfaction (SUS) <50 50-70 >70																														
		Malawi Satisfaction (SUS) <50 50-70 >70																														
NEST Qualified Technology?			No	No	No	No	No	No	No	No	No	No	No	No	No	No	No	No	No	No	No	No	No	No	No	No	No	No	No	No	No	No

Optimal | Minimal | Does not meet minimal | Unknown

Conductive Warmer

Evaluated against Target Product Profile (TPP) as reported by manufacturer

TPP Characteristic	Optimal Requirement	Minimal Requirement	A	B	C	D	E	F	G	H	I	J	K	L	M	N	O	P	Q	R	S	T	U	V	W	X	Y	Z	AA	BB	CC	DD	EE	FF
Intended Use	Treatment and prevention of hypothermia in neonates requireing thermal care																																	
Target Operator	For use in low- and middle-income countries by a wide variety of clinicans, including nurses, clinical officers, and pediatricians																																	
Target Population	Neonates (born at any gestational age and require ongoing care)																																	
Target Setting	Hospitals in low-resource settings																																	
Quality Management	ISO 13485:2016 Medical devices - Quality management systems																																	
Regulation	At least one of: CE marking, approved by US FDA or another strigent regulatory body of a founding member of IMDRF (e.g., Japan or Australia or Canada)																																	
Form Factor	Enclosed or not enclosed (no preference)																																	
Benchtop Measurement Accuracy Conductive Surface Temperature	Conductive Surface Temperature: Accuracy of control contact surface temperaute (measured) = ±0.1°C (and not exceeding 41°C)																																	
Temperature of Baby (required if servo-controlled)	Accuracy of baby's temperature: ±0.2°C																																	
Clinical Measurement Accuracy (Compare to another gold standard)	Known	Not required																																
Maximum CO2 Concentration (if Enclosed device)	0.50%																																	
Maximum Temperature (of the conductive surface)	40°C																																	
Humidification (if Enclosed device)	Humidity control for babies less than 1kg	None																																
Surface Temperature overshoot when the temperature control	1 °C																																	
Time to Indicate Accurate Temperature of baby	< 90 sec	< 5 min																																
Uniformity (if Enclosed, then uniformity of air) (If Not Enclosed, then uniformity of mattress)	Air Temperature: < 0.8°C (for enclosed only) Conductive Surface (for enclosed and not enclosed): High Heat: < 1°C Low Heat: < 0.5°C																																	
Alarm Characteristics	Visual and auditory																																	
Patient Interface	Interface is biocompatible and reusable	Interface is biocompatible																																
Patient Accessibility and Visibility	Patient is visible and accessible to healthcare worker																																	
Temperature Control	Based on infant's temperature and includes failsafe mode	Manual control and includes fail-safe mode																																
Operating Conditions	Describe in user manual how warming device is impacted by ambient temperatures in the operating environment																																	
Consumables (probes)	> 12 months before required	> 6 months before required																																
Instrument Pricing	< $500 ex-works	< $1,000 ex-works																																
Consumable Pricing	< $50 / year	< $100 / year																																
Power Source	Mains Power																																	
Power Consumption	< 250W maximum	< 800W maximum																																
Voltage	Model must match the voltage and frequency of the purchasing country's local power grid (e.g., 110-120 VAC at 60 Hz or 220-240 VAC at 50 Hz)																																	
User Manual	User manual and additional training materials (checklists, videos, guides) in at least one national official language for the country of intended use. Attached to device with labels and markings where possible.	User manual provided in at least one national official language																																
Warranty	5 years	1 year																																
Decontamination	Easy to clean with common disinfecting agents																																	
		Purchased for evaluations?	No	No	No	No	No	No	No	No	No	No	No	No	No	No	No	No	No	No	No	No	No	No	No	No	No	No	No	No	No	No	No	No
Technical	Does device meet all minimal performance characteristics from TPP?	Verified by lab evaluations on n = 2 devices by 3 unique users	Did not meet required specifications when evaluated against the TPP																															
Environmental	Is device rugged, durable, easy to maintain and repair?	Heat																																
		Humidity																																
		Dust																																
		Power																																
Usability	Can doctors and nurses use the device with minimal training?	HOU Satisfaction (SUS) <50 50-70 >70																																
		MW Satisfaction (SUS) <50 50-70 >70																																
		NEST Qualified Technology?	No	No	No	No	No	No	No	No	No	No	No	No	No	No	No	No	No	No	No	No	No	No	No	No	No	No	No	No	No	No	No	No

Optimal	Minimal	Does not meet minimal	Unknown

Respiratory Rate Apnea Monitor

Evaluated against Target Product Profile (TPP) as reported by manufacturer

TPP Characteristic	Optimal Requirement	Minimal Requirement	A	B	C	D	E	F	G	H	I	J	K	L	M	N	O	P	Q	R	S	T	U	V	W	X	Y	Z	AA	BB	CC	DD	EE	FF	GG	HH	II	JJ
Intended Use	To provide continuous monitoring of respiratory rate																																					
Target Operator	For use in low- and middle-income countries by a wide variety of clinicans, including nurses, clinical officers, and pediatricians																																					
Target Population	Neonates (born at any gestation age and require ongoing care)																																					
Target Setting	Hospitals in low-resouce settings, but, may be used in health facilites based on country guidelines	Hospitals in low-resource settings																																				
Quality Management	ISO 13485:2016 Medical devices - Quality management systems -- Requirements for regulatory purposes																																					
Regulation	At least one of: CE marking, approved by US FDA or another strigent regulatory body of a founding member of IMDRF (e.g., Japan or Australia or Canada)																																					
Apnea Detection	Detect periods of central apnea exceeding 20s duration (at 0)																																					
Respiratory Rate Accuracy	+/-2 bpm	+/- 5 bpm																																				
Respiratory Rate Range	0-100 bpm																																					
Alarm	Visual and auditory	An alarm (visual or auditory)																																				
Patient Interface	Interface is biocompatible and reusable	Interface is biocompatible																																				
Respiratory Rate Alarm Limits	Automatically adjust based on patient age	30-60 bpm																																				
Apnea Intervention	Yes	No																																				
Instrument Pricing	<$100 ex-works	<$250 ex-works																																				
Power Source	Mains with rechargeable battery																																					
Battery	Rechargeable battery, >24hrs on a single charge	Rechargeable battery, >6hrs on a single charge																																				
Voltage	Model must match the voltage and frequency of the purchasing country's local power grid (e.g., 110-120 VAC at 60 Hz or 220-240 VAC at 50 Hz)																																					
User Instructions	User manual and additional training materials (checklists, videos, guides) in at least one national official language for the country of intended use. Attached to device with labels and markings where possible.	User manual provided in at least one national official language																																				
Warranty	5 years	1 year																																				
Decontaimination	Easy to clean with common disenfection agents																																					
		Purchased for evaluations?	No	No	No	No	No	No	No	No	No	No	No	No	No	No	No	No	No	No	No	No	No	No	No	No	No	No	No	No	No	No	No	No	No	No	No	No

Technical	Does device meet all minimal performance characteristics from TPP?	Verified by lab evaluations on n = 2 devices by 3 unique users	Did not meet required specifications when evaluated against the TPP
Environmental	Is device rugged, durable, easy to maintain and repair?	Heat	
		Humidity	
		Dust	
		Power	
Usability	Can doctors and nurses use the device with minimal training?	HOU Satisfaction (SUS) <50 50-70 >70	
		MW Satisfaction (SUS) <50 50-70 >70	

			A	B	C	D	E	F	G	H	I	J	K	L	M	N	O	P	Q	R	S	T	U	V	W	X	Y	Z	AA	BB	CC	DD	EE	FF	GG	HH	II	JJ
		NEST Qualified Technology?	No	No	No	No	No	No	No	No	No	No	No	No	No	No	No	No	No	No	No	No	No	No	No	No	No	No	No	No	No	No	No	No	No	No	No	No
